# Therapy-based allied health delivery in residential aged care, trends, factors, and outcomes: a systematic review

**DOI:** 10.1186/s12877-022-03386-9

**Published:** 2022-08-28

**Authors:** Isabelle Meulenbroeks, Magdalena Z. Raban, Karla Seaman, Johanna Westbrook

**Affiliations:** Australian Institute of Health Innovation, Macquarie University, Level 6, 75 Talavera Road, North Ryde, 2122 Australia

**Keywords:** Residential aged care, Allied health services, Therapy, Systematic review

## Abstract

**Background:**

Allied health professionals in residential aged care facilities (RACFs) make important contributions to the physical and mental wellbeing of residents. Yet to date, health services research in RACFs has focused almost exclusively on nursing disciplines. This review aims to synthesise the current evidence on allied health services in RACF; specifically, how therapy-based allied health is delivered, what factors impact the quantity delivered, and the impact of services on resident outcomes and care quality.

**Methods:**

Empirical peer-reviewed and grey literature focusing on allied health service delivery in RACFs from the past decade was identified through systematic searches of four databases and over 200 targeted website searches. Information on how allied health delivered, factors impacting service delivery, and impact on resident outcomes were extracted. The quality of included studies was appraised using the Mixed Methods Appraisal Tool (MMAT) and the AACODS (Authority, Accuracy, Coverage, Objectivity, Date, Significance) checklist.

**Results:**

Twenty-eight unique studies were included in this review; 26 peer-reviewed and two grey literature studies. Sixteen studies discussed occupational therapy and 15 discussed physiotherapy, less commonly studied professional groups included dieticians (*n* = 9), allied health assistants (*n* = 9), and social workers (*n* = 6). Thirteen studies were assigned a 100% quality rating. Levels of allied health service provision were generally low and varied. Five studies examined the association between system level factors and allied health service provision, and seven studies examined facility level factors and service provision. Higher levels of allied health provision or access to allied health services, specifically physiotherapy, occupational therapy, and nutrition, were associated with reduced falls with injury, improved care quality, activities of daily living scores, nutritional status, and meal satisfaction in five studies.

**Conclusion:**

Evidence on how allied health is delivered in RACFs, and its impact on resident health outcomes, is lacking globally. While there are some indications of positive associations between allied health staffing and resident outcomes and experiences, health systems and researchers will need commitment to consistent allied health data collection and health services research funding in the future to accurately determine how allied health is delivered in RACFs and its impact on resident wellbeing.

**Supplementary Information:**

The online version contains supplementary material available at 10.1186/s12877-022-03386-9.

## Introduction

Residential aged care facilities (RACFs) provide long-term care for people, generally older adults, who are no longer able to live in their own homes. Internationally, these are also known as nursing homes/care homes or long-term care facilities. RACFs provide aid with activities of daily living such as showering, meals, and mobility, and provide medical care for chronic conditions.

Appropriate staffing in RACFs is important; research suggests that there is a positive association between nursing staff ratios and resident care quality and health outcomes [1–3]. However, many RACFs internationally reportedly operate with low nurse to resident ratios and often rely on personal care workers, who have minimal training requirements, to deliver complex care [4, 5]. Minimum staff ratios and reporting are mandated in some health systems such as in Canada, Germany, and the United States (US) [6]. However, the majority of nations don’t set minimum standards [7] and even when set, they may not be enforced or are criticised as still being too low to meet resident needs [8].

Research into staffing in RACFs lags behind research in hospital-based settings, and has been described as limited and of overall poor quality [9]. The research that is available on the aged care workforce and minimum standards set, almost exclusively focuses on the nursing discipline neglecting other important multidisciplinary team members. To ensure that the workforce can provide high-quality care now and in the future research on all professional groups providing care is needed.

Allied health professionals are essential team members in the care of older adults. Allied health is an umbrella term. While there is no unanimously agreed definition, it is often used to describe disciplines that fall outside of medicine and nursing professions and includes physiotherapy, occupational therapy, dietetics, optometry, pharmacy, audiology, radiology, and podiatry. In RACFs allied health professionals can help maintain residents’ independence, prevent falls, manage common symptoms such as swallowing difficulties, chronic pain, and malnutrition, and play an important role in educating the aged care workforce [10–13]. Due to the disparate nature of allied health professions, this review focuses on therapy-based allied health professions–professions that provide care that is not directly pharmacological, technological, or imaging related–for example physiotherapy, occupational therapy, social work, and speech pathology. The division of allied health into therapy and scientific disciplines has been used previously in Australian workforce reports [14].

Exactly how allied healthcare is provided to aged care residents, by whom, and the ideal service levels to maximise positive outcomes for residents is unclear. Allied health is a diverse group of professionals; education, practice, evaluation, oversight, and registration requirements for each professional group varies significantly, even within a single health system [15–17]. Such fragmentation means that data are inconsistently collected and overall data on allied health, when available, are often collected by individual professional groups and organisations [16]. Despite the complexity, synthesising information on allied health in aged care is essential to identifying workforce capacity, inform future workforce planning, and to ensure residents receive high quality care [18, 19]. This review aims to fill this evidence gap by synthesising evidence on i) how therapy-based allied healthcare is delivered in RACFs globally, ii) factors associated with levels of allied health service provision, and iii) resident care quality and health outcomes associated with allied health staffing.

## Methods

### Protocol

This review followed a registered protocol (CRD42021266141). The study design was informed by the Preferred Reporting Items for Systematic Reviews and Meta-Analyses (PRISMA) checklist [20] and the Joanna Briggs Institute Manual for Evidence Synthesis [21].

The registered protocol outlined an aim to review how allied health was delivered during the COVID-19 pandemic. After initial searches and screening, no relevant literature was found. The search strategy and aim were subsequently expanded to cover the last ten years to gain an understanding of how allied health is currently delivered in RACFs, trends, and associated outcomes.

### Search strategy

Four databases, Medline, Scopus, CINAHL, and EMBASE, were systematically searched in June 2021. Themes used in the search strategy included “allied health”, “residential aged care”, and “workforce/staffing”. All searches were limited to the last ten years and articles published in English. The search strategy (Additional file 1) was developed in consultation with a medical librarian, was informed by previous reviews in the field, and received feedback from the research team. Additional articles were sought by manually searching the reference lists of included studies, that is citation searching.

The peer-reviewed search strategy themes were applied to search the grey literature. Over 200 targeted searches were conducted in the Google Advanced, government, think tank, allied health, and aged care websites of English-speaking countries (Additional file 2). While searching the grey literature, the first ten articles of each search were screened for potential relevance. Searches were recorded in a purpose designed Excel spreadsheet.

Following the execution of the search strategies and initial screening, the research team chose to narrow the included allied health professions to therapy-based care as some care, outcomes and trends, arising from services such as radiography and pharmacy, were too diverse to synthesise with therapy-based care. The articles retrieved were manually screened for therapy-based allied health professional groups.

### Inclusion and exclusion criteria

Peer-reviewed and grey literature articles were included if they were published in the last ten years, investigated therapy-based allied health professionals or allied health assistant service delivery in a RACF, and collected data on how allied healthcare is delivered (i.e., workforce characteristics, frequency, intensity, or access to allied healthcare), factors associated with allied healthcare delivery (e.g., facility size, funding, patient characteristics), or impact of allied healthcare services on resident health or care quality. Articles were excluded if published in another language other than English, if they were reviews or non-empirical studies, or studied a specific clinical intervention.

In this review, therapy-based allied health included chiropractors, dietetics, exercise physiology, music therapy, occupational therapy, osteopathy, physiotherapy, psychology, podiatry, social work, speech pathology, diversional therapists, recreational therapists, lifestyle officers, cultural officers, and related allied health assistants. This definition was modified from local government and peak organisation definitions to be inclusive the terms used to describe therapeutic allied health disciplines internationally [14].

### Screening

References, including title and abstracts, were imported into Rayyan [22], a web-based artificial intelligence platform that supports manual screening by scanning and highlighting terms relevant to inclusion and exclusion criteria. Peer-reviewed articles underwent a two-step screening process: title/abstract and full-text screening. As grey literature articles often do not have an abstract available, articles proceeded directly into the full-text screening. Ten percent of articles at each stage were screened by three reviewers (IM, KS, MR) to assess inter-rater reliability and application of the inclusion/exclusion criteria. Inter-rater reliability between reviewers was good [23]; title/abstract screening k = 0.60 (95% CI 0.60–0.60), *p* < 0.01, full-text screening k = 0.67, (95% CI 0.66–0.68) *p* < 0.01. Conflicts between reviewers and screening uncertainties were discussed at regular team meetings. The remaining 90% of articles were screened by one reviewer (IM).

### Data extraction

Data were extracted from included articles using purpose designed Excel spreadsheets tailored to the grey and peer-reviewed literature. Extracted data included, but was not limited to details on author, country, study design, data sources, participants, characteristics of allied health professionals, allied health service delivery, and health or care quality outcomes. Data extraction was completed by one reviewer (IM). The data extraction sheet and extracted data were reviewed by the research team to ensure completeness and quality in data extraction (KS, MR).

### Quality appraisal

Peer-reviewed study quality was appraised using the Mixed Methods Appraisal Tool (MMAT) [24] as it can be used to appraise the multiple study designs. The MMAT results were summarised as a percentage of “yes” judgements in each of the five domains [25].

The quality of grey literature articles was appraised using the AACODS checklist (Authority, Accuracy, Coverage, Objectivity, Date, Significance) [26]. The AACODS checklist was selected in addition to the MMAT tool as it can be used across peer-reviewed and grey literature. AACODS results were summarised as a percentage of “yes” judgements in each of the six domains. Two independent reviewers applied the MMAT and AACODS checklist to each article as appropriate (IM, KS, MR). Conflicts in quality appraisal were discussed and resolved in regular team meetings. Studies of overall poor quality were not excluded from the review due to the limited literature.

### Synthesis

Publications arising from the same author group with a similar population and focus were grouped together in unique study groups to minimise overrepresentation of some populations and results. For example, publications related to an Australian RACF workforce census, conducted every four years, were grouped into one unique study group. Hence at times multiple references are provided when referring to one study. All results were narratively synthesised to address the study aims: how allied health is delivered, factors associated with allied health delivery, and associated resident outcomes.

## Results

**Fig. 1:**
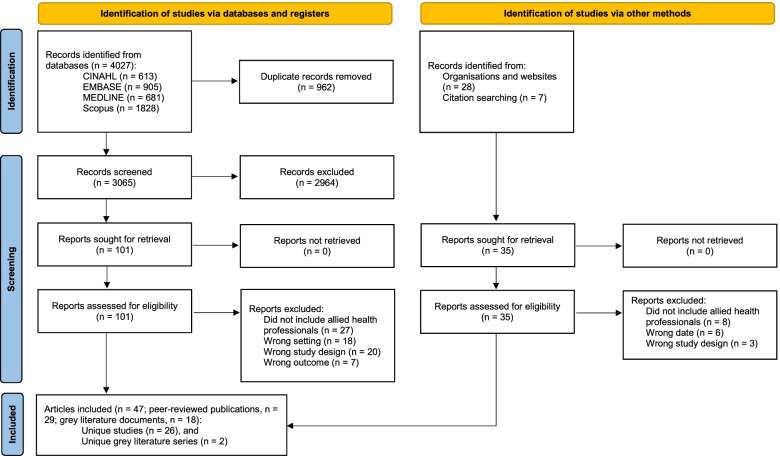
PRISMA diagram

Database searching retrieved 4027 articles (CINAHL: 613, EMBASE: 905, Medline: 681, Scopus: 1828). After removing duplicates, 3065 articles remained. All underwent title/abstract screening and subsequent full-text screening if appropriate. Absence of allied health professionals and settings other than RACFs were common reasons for exclusion in this process. The grey literature searching and citation searching retrieved 28 and 7 articles respectively. In total, 28 studies were included in the review; 26 peer-reviewed and 2 grey literature unique studies arising from 47 articles (Fig. 1).

Most peer-reviewed studies were conducted in the US (*n* = 12), followed by Canada (*n* = 6), the Netherlands and/or Germany (*n* = 3), and Australia (*n* = 2). One study was conducted in each of the following: Ireland, Norway, South Korea, Sweden, Italy, and the United Kingdom (UK) (Table 1). Studies most frequently used questionnaires (*n* = 13) to analyse allied health service delivery in RACFs; administrative data (*n* = 10), patient medical records and registries (*n* = 3), and interviews (*n* = 1) were utilised less for analysing allied healthcare services.

**Table 1:** Study characteristics

Study	Country	Description	Data source	Sample	AHPs	Quality
***Peer-reviewed studies***						
Bennett (2019) [27]	Australia	Profile SP workforce practices and characteristics	Questionnaire	145 SP	SP	80%
Bern-Klug (2013) [28]	US	Detail SW involvement in specific activities in RACFs	Questionnaire	1071 RACFs	SW, SWA	60%
Bhuyan (2017) [29]	US	Explore the provision of rehabilitative services and organisational determinants in RACFs	Questionnaire	31,134 RACFs	PT, OT	100%
Bonaccorsi (2015) [30]	Italy	Describe processes and outcome indicators of nutritional care in RACFs	Questionnaire	67 RACFs; 2395 residents	D	80%
Buddingh (2013) [31]	Canada	Explore rehabilitation practices and barriers for residents with dementia post hip fracture in RACFs	Questionnaire	42 AHPs	PT, OT, AHA	60%
Burger (2017) [32]	Germany	Describe nutritional care practises in RACFs	Questionnaire	541 RACFs	D	100%
Enam (2013) [33]	US	Understand OT practice patterns in sleep management in RACFs	Questionnaire	113 OT and OTAs	OT, OTA	60%
Engh (2021) [34]	Norway	Explore management of dysphagia in RACFs	Questionnaire	121 RACFs	OT, PT, SP, SW, N	60%
Giesbracht (2012) [35]	Canada	Identify the extent of the need for wheelchair seating assessment in RACFs	Medical records	11 RACFs; 263 residents	OT	60%
Hirdes (2011) [36]	Canada	Examine person level clinical data in RACFs	Administrative data	54 RACFs; 129,168 residents	PT, OT, SP, Psych, RT	100%
Hsu (2016) [37]	Canada	Present longitudinal changes in staffing levels in RACFs	Administrative data	627 RACFs	PT, OT, other therapy/ recreation staff (not specified)	100%
Hurley (2017) [38]	Ireland	Explore current diabetes management in RACFs	Questionnaire and interviews	33 RACFs	D, P	80%
Kinley (2014) [39]	England	Identify care provided to dying residents in RACFs	Medical records	38 RACFs; 2444 residents	AT, D, MT, PT, SP, SW, OT, P, Psych	80%
Koenig (2011) [40]	US	Explore RACF administrators’ perspectives of SW	Interview	13 administrators	SW, SWA	100%
Lee (2011) [41]	South Korea	Explore factors which impact resident performance in activities of daily living	Administrative data	358 RACFs; 14,369 residents	PT	100%
Liu (2015) [42]	US	Describe the use of music therapy by hospice patients	Administrative data	4804 hospice patients	MT	100%
Livingstone (2019, 2020, 2021) [43–45]	US	Examine the impact of AHP staffing on care quality and characterise RACFs associated with staffing patterns	Administrative data	12,352 RACFs	PT, PTA, OT, OTA, SP	100%
McArthur (2015) [46]	Canada	Describe the proportion- and characteristics of residents receiving AHP services	Administrative data	87,869 residents	PT, OT	100%
McArthur (2018) [47]	Canada	Examine the impact of policy change on AHP staffing levels, activities of daily living, and care quality in RACFs	Administrative data	589 RACFs	PT, OT, SP	100%
Roberts (2018, 2017) [48, 49]	US	Understand trends in social service department staffing in RACFs including the impact of rurality	Administrative data	15,936 RACFs	SW, SWA	100%
Skinnars (2017) [50]	Sweden	Explore indicators associated with nutritional status and satisfaction in RACFs	Questionnaire and national registry	162 municipalities	D	80%
Stargatt (2017) [51]	Australia	Explore access to psych services in RACFs	Questionnaire	90 health professionals	SW, OT, Psych, DT	40%
Sterke (2021) [52]	Netherlands	Describe PT practice in RACFs	Questionnaire	46 PTs	PT	60%
Temkin-Green (2019) [53]	US	Identify trends in therapy provision, particularly high intensity therapy, at end-of-life, and associated RACF characteristics	Administrative data	647 RACFs; 5591 residents	PT, OT, SP	100%
Tyler (2013) [54]	US	Track staffing trends in RACFs	Administrative data	14,331 RACFs	PT, AHA, OT	80%
Van Nie-Visser (2011) [55]	Germany, Netherlands	Compare nutritional care, structural indicators, and risk of malnutrition in RACFs between two health systems	Questionnaire	151 RACFs; 10,771 residents	D	80%
***Grey literature studies***						
StewartBrown (2017, 2018, 2019, 2020, 2021) [56–69]	Australia	Quarterly financial report on the aged care sector	Questionnaire, administrative data	135–1267 RACFs	D, PT, SP, OT, P, lifestyle officers, DT, AHA, other (not specified)	66.7%
Department of Health, Australian Government (2013, 2017, 2020) [70–72]	Australia	Aged care workforce census	Questionnaire	1329–2952 RACFs	D, DT, PT, SW, SP, OT, P, Psych, AHA, EP, Other (not specified)	100%

*AHP* Allied health professional, *AHA* Allied health assistant, *AT* Art therapists, *D* Dieticians, *DT* Diversional therapists, *EP* Exercise physiologist, *MT* Music therapists, *N* Nutritionists, *OT* Occupational therapists, *OTA* Occupational therapy assistants/aides, *P* Podiatry, *Psych* Psychologists/mental health services, *PTA* Physiotherapy assistants/aides, *RT* Recreational therapy, *SP* Speech pathologists, *SW* Social work, *SWA* Social work assistants/unregistered/untrained social workers

All included grey literature studies were Australian (*n* = 2). One study, a financial report on Australian RACFs, published quarterly, used questionnaires and administrative data while the other, a workforce census conducted every four years, used a questionnaire. Details on methods and definitions of allied health professions included were often missing in these reports.

Occupational therapy (*n* = 16) [29, 31, 33–37, 39, 43–47, 51, 53, 54, 56–72] was the most frequently explored professional group in included studies followed by physiotherapy (*n* = 15) [29, 31, 34, 36, 37, 39, 41, 43–47, 52–54, 56–72], speech pathology (*n* = 10) [27, 28, 34, 36, 39, 43–45, 47, 53, 56–72], nutrition and dietetics (*n* = 9) [30, 32, 34, 38, 39, 50, 55–72], allied health assistants (*n* = 9) [28, 31, 33, 40, 43–45, 48, 49, 54, 56–72], social workers (*n* = 6) [34, 39, 40, 48, 49, 51, 70–72], art therapy, music therapy, and/or diversional therapy (*n* = 6) [36, 37, 39, 42, 51, 56–72], psychologists/mental health professionals (*n* = 4) [36, 39, 51, 70–72], and podiatrists (*n* = 4) [38, 39, 56–72].

### Quality appraisal results

Twelve peer-reviewed studies achieved 100% in the MMAT. Common reasons for lower scores were high risk of non-response bias (*n* = 7) [27, 31, 33–35, 51], non-representative samples (*n* = 5) [31, 33, 35, 39, 51], and use of unvalidated measures (*n* = 2) [28, 51]. Only one study was assessed to be of poor quality (≤ 40%) [51].

Grey literature studies generally scored well on the AACODS assessments. One study lost points in the authority and coverage domain due to missing or unknown expertise, authority in the field, and parameters of allied health covered. Quality appraisal results are presented in detail in Additional file 3.

### How allied health is delivered

#### Allied health workforce

In Australia, occupational therapists (37%, *n* = 783), physiotherapists (44%, *n* = 2874), podiatrists (84%, *n* = 928) [56, 70, 72], dieticians (81%, *n* = 787), exercise physiologists (77%, *n* = 192), psychologists 87%, *n* = 185), social workers (61%, *n* = 219), and speech pathologists (86%, *n* = 692) were commonly employed part-time as subcontractors [72]. Allied health assistants (75%, *n* = 2238) and diversional therapists (71%; *n* = 2258) were the only allied health workers that were more commonly employed by facilities in permanent part-time roles, rather than as a subcontractor [72]. In a 2020 report allied health assistants made up 22% (*n* = 2992) of all allied health positions in Australian RACFs however the number of all allied health workers in the sector has been declining from 2013 [70, 72].

Surveys demographics in Canada, the US, and the Netherlands suggest that the workforce, specifically occupational therapists [31], social service workers [28, 40], and physiotherapists [31, 52, 33], is predominately female [31], holds a university degree [28, 31, 40], and is experienced in aged care [28, 33, 52]. In Canada and the Netherlands, occupational therapists, and physiotherapists were predominately part time [31, 52] while the US occupational therapists and assistants were more likely to be full time (72%, *n* = 101) [33].

#### Allied health roles

Information on the roles performed by allied health in RACFs was limited. Mobility was an important aspect of physiotherapy care in Dutch [52] and Canadian [31] studies. Dutch physiotherapists also reported that falls prevention, advice on mobility transfers, and management of pressure ulcers were a common aspect of their role [52]. US social workers reported that common tasks in their role included attending quarterly care plan meetings and providing emotional support to families [28]. One US study found that the scope of occupational therapy practice, specifically sleep interventions, in RACFs is limited by lack of skills, resources, and facility culture [33].

Role substitution was discussed in two studies. In RACFs in Norway swallow assessments were conducted by speech therapists (7.4%), occupational therapists (6.6%), physiotherapists (3.3%), nutritionists (1.6%) and overall were more likely to be conducted by nursing staff (92.6%; *n* = 121) [34]. An Australian survey found that residents with depression were just as likely to be referred to an occupational therapist as a psychologist [51].

#### Allied health service delivery

##### Proportion of residents receiving therapy

Overall, six studies measured the proportion of residents receiving therapy in RACFs (Table 2). Globally, the proportion of residents receiving physiotherapy in RACFs ranged from 5.6% to 56.6% [36, 41, 46, 47] in a seven-day period. Both highest [47] and lowest [36] proportions were reported in Canada across different provinces. Data on the proportion of residents receiving occupational therapy, speech pathology, recreational therapy, and mental health was only available in Canadian studies and ranged from 1–21.9% [36, 47], 0.2–4.4% [36], 9.6–53.1%, and 7.3% respectively [36].

**Table 2:** Proportion of residents who received allied health over a specified period, grouped by period

AHP ^a^	Country	Sample size (n)	Data collection period	Proportion of residents who received AHP services (%)
***7 days***				
Physiotherapy	Canada^b^	129,168 [36]; 589^c^ [47]	2009–2010 [36]; 2013 [47]	5.6 [36]–56.6 [47]
	Korea	14,369	2008	22.5 [41]
Occupational therapy	Canada^b^	129,168 [36]; 589^c^ [47]	2009–2010 [36]; 2013 [47]	1.0 [47]–21.9 [36]
Speech pathology	Canada	129,168	2009–2010	0.2–4.4 [36]
Recreational therapy	Canada	129,168	2009–2010	9.6–53.1 [36]
***90 days***				
Mental health services^d^	Canada	129,168	2009–2010	0–7.3 [36]
***Last 30 days of life***				
Physiotherapy, occupational therapy, and speech pathology	US	55,691	2012–2016	13.3 [53]
***Last six months of life***				
Physiotherapy	England	2318	2008–2011	12 [39]
Occupational therapy	England	2318	2008–2011	1 [39]
Speech pathology	England	2321	2008–2011	8 [39]
Social work	England	2320	2008–2011	9 [39]
Dietetics	England	2319	2008–2011	12 [39]
Podiatry	England	2319	2008–2011	22 [39]
Alternative/creative therapist	England	2317	2008–2011	0.7 [39]
Mental health services ^d^	England	2317	2008–2011	11 [39]
***Entire admission***				
Dietician	Germany	1305^e^	2008–2009	9.3 [55]
	Netherlands	1521^e^	2008–2009	36.7 [55]

^a^ AH = Allied health profession

^b^ Two studies presented values of allied health professional group and country, highest and lowest values of each are provided

^c^ 589 is the number of RACFs, all other values are resident sample size

^d^ Professional group not specified; e malnourished residents only

The proportion of residents receiving therapy in specific populations within RACFs was the focus of three studies; two end-of-life specific [39, 53] and one in malnourished residents [55]. In the UK, the proportion of residents receiving therapy in the last six months of life from a single allied health profession ranged from 0.7% for alternative therapy to 12% for physiotherapy services (Table 2) [39]. A US study concluded that the proportion of residents receiving therapy (inclusive of physiotherapy, occupational therapy, and speech pathology) at the end-of-life (last 30 days) increased in the four-year study period from 11.6% to 13.3% (*n* = 55,691). The proportion of residents receiving ultrahigh therapy, defined as > 720 min of therapy a week, also rose from 4.4% to 7.3% at the end-of-life (last 30 days) (*n* = 55,691), this was particularly concentrated in the last two weeks of life [53]. The proportion of residents who received a visit from a dietician varied. In the Netherlands 36.3% of malnourished residents were seen by a dietician during their admission compared to 9.3% in Germany (Table 2) [55].

##### Intensity of allied health services

Information on hours per resident per day (HPRD) for specific disciplines was available in three Australian or US-based studies. Where information is available, HPRD was consistently higher in the US (Table 3). The total sum of allied health HPRD was available for Australia and Canada, where Australian residents received 0.03 HPRD more therapy than Canadian residents.

**Table 3:** Availability of allied health professions, grouped by profession

AHP^a^	Type of engagement^b^	Country	Sample size (number of RACFs)	Data collection period	Proportion of sample size providing or having access to AHP service (%)
Physiotherapy	Employed	US	31,134	2010	43.9 [29]
		Norway	91	2020	37.4 [34]
Occupational therapy	Employed	Australia	82	2015	43.0 [51]
		US	31,134	2010	40.0 [29]
		Norway	94	2020	53.2 [34]
Speech pathologists	Employed	Norway	78	2020	7.7 [34]
Social work	Employed	US	15,936	2009–2012	89.1^c^ [49]
		Australia	82	2015	17 [51]
		Norway	77	2020	6.5 [34]
Social work assistants	Employed	US	15,936	2009–2012	57.2^c^ [49]
Therapy assistant	Employed	Canada	37	2010	84.0 [31]
Nutrition and dietetics	Employed	Norway	79	2020	6.3 [34]
	Access	Germany	541	2014	42.3 [32]
		Ireland	33	2013	82 [38]
		Sweden	1154	2014	75.5 [50]
		Italy	67	2012	88 [30]
Podiatry	Access	Ireland	32	2013	97 [38]
Diversional therapy	Employed	Australia	88	2015	73.9 [51]
Psychology	Employed	Australia	81^d^	2015	13.6 [51]

^a^ AHP = Allied health profession

^b^ Engagement indicates whether the facility employed the specified allied health profession or whether that had access to the profession through internal and/or external staff

^c^ longitudinal measure, all other measures were collected a cross-sectionally

^d^ survey respondents may or may not be from individual RACFs

The HPRD of allied health in Australia has increased from 6.6 min in June 2016 [56–69] to 15 min of care per resident per day in 2020 [56]. However, the definition of allied health used to collect data had changed over this period [56–69]. In the US, physiotherapy and occupational therapy HPRD has increased in free standing RACFs but declined in hospital-based facilities [54].

Intensity of allied health service provision was presented as allied health staff ratios in two studies. In a US study a sample of 1071 RACFs on average hired 1 full-time social service employee per 89.3 beds [28]. In one Canadian city, a sample of 11 RACFs, employed 0.68 occupational therapists per 100 residents [35].

Intensity of allied health services was also measured in the number of and/or length of visits in five studies. In South Korea, of those receiving physiotherapy treatment, 5–7 sessions per week was the most common frequency (66.5%; *n* = 3230) [41]. One Canadian study found that of residents who received physiotherapy, on average they received 49.1 min over 2.9 days (*n* = 589 RACFs) [47]. Another Canadian study simply reported that occupational therapists saw residents with hip fracture less frequently than physiotherapists, but session times were longer; quantities were not provided [31]. In the US, a large hospice provider provided on average 11.49 music therapy visits to hospice patients in RACFs (*n* = 2930), totalling 7.5 h, at 38 min each, of care from a music therapist [42]. In the UK residents received between 0–56 visits from an allied health professional in the last six months of life. All allied health professional groups measured on average did not visit residents at end-of-life, expect physiotherapy where residents on average received one visit [39].

##### Allied health service availability

Access to or employment of allied health professionals was used to measure allied health service delivery in eight studies. Globally, 37.4–43.9% of RACFs provided a physiotherapy service [29, 34], 40–53.2% provided and occupational therapy service [29, 34], 6.5–89.1% provided a social worker service [34, 49], and nutrition and/or dietetics was accessible in 42.3–88% of RACFs [30, 32]. Information on access to or employment of psychologists [51], diversional therapists [51], speech pathologists [34], and podiatry [38] were only available in individual studies (Table 4).

**Table 4:** Mean hours per resident day (HPRD) receiving allied health services

	**Australia** (2020, *n* = 331)	**US** (2013–2016; *n* = 12,352) [43–45]; (2009–2015; *n* = 15,936) [48, 49]	**Canada** (1996–2011; *n* = 627)
Physiotherapy	0.09 [56]	0.10 [43–45]	
Occupational therapy	0.01 [56]	0.09 [43–45]	
Social work		0.041 [48, 49]	
Physiotherapy assistants	0.01^a^ [56]	0.10 [43–45]	
Occupational therapy assistants		0.09 [43–45]	
Social work assistants		0.076 [48, 49]	
Speech pathology	< 0.01 [56]	0.06 [43–45]	
Podiatry	0.01 [56]		
Dietetics	< 0.01 [56]		
Lifestyle officers	0.12 [56]		
Diversional therapists	0.01 [56]		
Other	0.01^a^ [56]		
Total	0.25 [56]		0.22^a^ [37]

^a^ specific discipline/s not specified

In Canada, 84% of survey respondents reported that their facility hired a therapy assistant [31]. In the same US study paraprofessional staff were hired to deliver social care in 57% of facilities (Table 4) and were the only source of social care in 11–18% of RACFs [40, 49].

### Factors influencing allied health services

#### System level factors associated with allied health service delivery

A total of five studies examined the association between system level factors and allied health service delivery. One study investigated the impact of funding on allied health service delivery. Canadian funding changes, in a sample of 589 RACFs, decreased the proportion of residents receiving occupational therapy from 2.5 to 1% and physiotherapy from 84.6% to 56.6%, speech pathology remained consistent at 0.3% [47].

Four studies reported the impact of location on allied health services. Physiotherapy service provision was higher in certain provinces in a US and Canadian study [43, 46]. Occupational therapy services were also associated with state in the US [43]. Rurality impacted allied health services in two US-based studies; residents in urban facilities were more likely to receive therapy at end-of-life [53], while rural facilities hired fewer full time equivalent qualified social workers and overall provided fewer HPRD of social service staff [48, 49] (Additional file 4).

#### Facility level factors associated with allied health service delivery

A total of 7 studies examined the association between facility level factors and allied health service delivery. Four US studies looked at the impact of facility size on allied health service provision. Three studies reported that services provided by physiotherapists, physiotherapy assistants and aides, occupational therapists [29], dieticians, interprofessional nutritional teams [32], and qualified social workers [49] were more likely in larger facilities. Conversely, other studies reported that physiotherapy, occupational therapy, and overall social service staffing was highest is smaller facilities [45, 48]. There was no association between facility size and occupational therapy assistant and aide staffing in one study [44].

The impact of RAC ownership on therapy services was explored in five studies. Studies from the US reported that for-profit facilities provided more physiotherapy and occupational therapy care [29], including high intensity therapy at end-of-life [53], and were more likely to hire allied health assistants to deliver social services (rather than qualified social workers) when compared to non-profit facilities [49]. Conversely two studies, from the US and Canada, reported non-profit providers provided more therapy per resident compared to for-profit [37, 43, 45]. The there was no association between ownership and physiotherapy assistant and occupational therapy aide HPRD in one US study [43, 45].

The impact of Medicare and Medicaid certification, a characteristic specific to US studies, on allied health service delivery was examined in four studies. Physiotherapy, occupational therapy, respective assistants [29, 43], ultra-high therapy at end-of-life [53], and use of interprofessional team to deliver social care [49] was more common in facilities that had a higher proportion of Medicare residents. Medicaid certification was associated with lower numbers of qualified social workers [49]. Medicaid was also associated with lower hours of physiotherapy and occupational therapy staffing in one study [43], but higher service delivery in others [29, 53]..

Three US studies reported on overall service utilisation and allied health services. Physiotherapy and occupational therapy services were more likely in facilities which had a high ratio of personal care assistants [29] and in facilities with higher service utilisation [43]. A higher ratio of licensed nurses reduced the likelihood of ultra-high therapy at the end-of-life [53].

Three US studies reported on the association between therapy staffing and occupancy rates. Lower RACF occupancy rates were associated with high physiotherapy and occupational therapy staffing in one study [43, 44] but no association in two others [29, 53].

Two US studies reported on the impact of hospital-based location on allied health HPRD. Hospital-based facilities had higher physiotherapy, occupational therapy, and qualified social worker [43, 44] HPRD but had lower use of paraprofessionals and interprofessional teams to deliver social services [49].

Two US studies reported the impact of case mix on allied health staffing. Higher acuity was associated with higher physiotherapy and occupational therapy staffing [43, 45], but was not associated with social work or social services staffing [49] (Additional file 4).

#### Resident factors associated with allied health service delivery

Two studies reported on resident characteristics and their association with receiving allied health services. In a Canadian study, physiotherapy services were more likely in younger residents, with a diagnosis of multiple sclerosis, Parkinson’s disease, pneumonia, fracture, self-rated ability to improve and were less likely in residents who were cognitively impaired [46]. In the US, the likelihood of therapy at end-of-life was higher in men and people from minorities [53]. Both studies agreed that therapy was more likely in residents who recently experienced an acute episode [46, 53] (Additional file 4).

### Association of allied health services with resident outcomes

#### Activities of daily living

Three studies investigated the relationship between physiotherapy and occupational therapy and performance in activities of daily living, they all suggest a positive association with professional staffing levels (Additional file 5). A Korean study found that more physiotherapy at baseline was associated with having improved activities of daily living scores at six months follow-up [41]. A Canadian study found that facilities that had a high proportion of residents not receiving physiotherapy scored poorer in activities of daily living measures and that higher intensity of physiotherapy may prevent a decline in these measures [47]. Physiotherapy and occupational therapy staff HPRD were associated with improvements in activities of daily living. A one-hour increase in physiotherapy and occupational therapy staffing improved activities of daily living measures by 2.9 and 3.7 points respectively [44, 45]. There was no association between activities of daily living and physiotherapy and occupational therapy assistants or aides [44, 45].

#### Falls

Two studies investigated the impact of physiotherapy and occupational therapy on falls measures (Additional file 5). A Canadian study found that, while a higher intensity of physiotherapy visits did not improve the proportion of residents who fell in the last 30 days, facilities that provided fewer physiotherapy visits had more falls [47].

In a US study, for every HPRD of physiotherapy and occupational therapy a falls measure (the percentage of residents experiencing one or more falls with major injury) declined by 1.1 and 0.8 percentage points respectively [44, 45]. However, higher HPRD of physiotherapy assistants were associated with an increase in the same falls measure. There was no association between physiotherapy aides and occupational assistant or aide HPRD and falls [44, 45].

#### Care quality

Two studies investigated the association between physiotherapy and occupational therapy, and measures of care quality. Both found an association with professional staffing levels; assistant staffing levels had no impact (Additional file 5). An increase of one occupational therapist to 100 residents in a Canadian study reduced the probability of wheelchair seating issues by 90% [35]. High occupational therapy and physiotherapy HPRD in the US were associated with increased likelihood of obtaining five stars in the Five Star Quality Rating System. Specifically, a one unit increase in occupational therapy and physiotherapy HPRD resulted in three- and two-times greater likelihood of achieving four or five stars in the quality measure respectively [44, 45]. However, occupational therapy and physiotherapy assistant or aide HPRD was not associated with care quality measures [44, 45].

#### Nutrition

One Swedish study investigated the association between allied health service delivery and nutrition-related outcomes. Having a food service dietician or clinical/community dietician was associated with high resident meal satisfaction, using an unvalidated questionnaire, and the availability of a clinical/community dietician increased the likelihood of residents being well nourished (Additional file 5) [50].

## Discussion

This review found that allied health service delivery in RACFs and the measures used to quantify use and impact varied significantly within and between health systems. Overall, it is unclear how allied health services are delivered in RACFs in any one health system. Additionally, factors associated with allied health service delivery in RACFs, such as profit and non-profit facilities, often showed contradicting associations across studies, limiting the generalisability of results. But the limited evidence available, suggests that allied health professional services may improve care quality, activities of daily living, falls, and nutrition [44, 45, 47, 50].

Overall allied health service provision is low, highly varied, and may not meet international recommendations. For example, total allied health service provision ranged from 15 min per resident per day in Australia [56, 73] to 13.2 min in Canada [37]. This service provision may be higher than other countries which do not report overall figures, such as Korea which only provides physiotherapy to 22.5% of residents [41]. Allied health staffing is also highly dependent on individual and facility factors; as there is limited evidence on specific intensities and skill mix on allied health services it is likely that individual facilities simply tailor services according to available funding and/or local directives, which can vary significantly based on factors such as size, locality, profit status, and certification. With such significant system variation, and often low levels of service, it seems unlikely that allied health services consistently meet broader international exercise or therapy targets, such as the European recommendation of twice week exercise programs lasting 35–45 min each [74] or Ontario’s more ambitious target of 36 min of allied health service delivery per resident per day [75]. Further research and policy work is urgently needed to determine the optimal levels and mix of therapy in RACFs, and incentives and models of care to consistently support its delivery.

Mandatory registration of allied health professions has previously been suggested as a solution to address inconsistent and limited data [19, 76]. However, practically, static measures of how many professionals are employed by facilities (e.g., Table 4) or are registered in a health system are not meaningful measures of service delivery; they do not quantify the amount of face-to-face care and depict allied healthcare delivery in rigid professional silos whereas in practice roles are substituted and care is delivered in teams. For example, in this review, swallow assessments were performed by speech pathologists, nurses, occupational therapists, and physiotherapists [34]; and mental healthcare was provided by a range of people including psychologists, occupational therapists, and even pastoral care [51]. RACFs in studies in this review also employed people ranging from tertiary educated and registered professionals to those without a formal education [28, 40], to allied health assistants without supervision [49], volunteers [29], and students [70] to deliver allied health services. To meaningfully quantify allied health services, all facilities should, as a minimum standard, record the volume of direct care provided by all staff.

In this review we found professional therapy staffing improved falls, independence, meal satisfaction, and care quality [41, 44, 45, 50]. While this is promising, these five studies–all with diverse outcome measures–do not provide a strong evidence-base to justify an increase in allied health staffing in RACFs. Significant further commitment to research on the impact of allied health services and appropriate future quality indicators is required [19]. As consistent national data on allied health services often does not exist, current opportunities for this research include consumer-research-provider partnerships and the use of employment data and electronic medical records. Further research should explore appropriate consumer-focused measures of allied health service delivery and outcomes in RACFs.

### Limitations

Some relevant articles may have been missed in the search strategy. Many articles found during citation searching did not mention allied health services in the keywords, title, or abstract, rather only occasionally in the full text. To account for this extensive time was spent hand searching aged care staffing literature.

All included grey literature arose from Australia. It may be that search engines used prioritised local literature. To address this, extra time was spent searching international government websites however all relevant international literature fell outside the included date range.

Quality appraisal scores were not used to limit included articles as the available body of literature was small. However, only one study had an overall rating of poor quality, and we do not believe including this study unduly influences our conclusions. Furthermore, we have pointed out the poor-quality study when presenting our results.  

## Conclusion

This review demonstrates that allied health service delivery is inconsistent within and between health systems; service delivery is impacted by nearly every variable studied—including funding, facility size, rurality, service utilisation, health status—and overall, is data poor. Positively, the few studies that studied outcomes found largely positive associations in independence, falls, care quality, and nutrition outcomes with higher allied health provision levels. To improve care quality in RACFs health systems should make efforts to collect data on allied health professions and increase allied health service research practices.

## Supplementary Information

**Additional file 1:** Medline OVID search strategy.

**Additional file 2:** Websites searched in grey literature search strategy.

**Additional file 3:** MMAT scores.

**Additional file 4:** Factors associated with allied health service delivery.

**Additional file 5:** Outcomes associated with allied health staffing.

## Data Availability

All data generated or analysed during this study are included in this published article and its supplementary information files.

## Abbreviations

AACODS: Authority, Accuracy, Coverage, Objectivity, Date, Significance
AHP: Allied health professional
MMAT: Mixed method appraisal tool
RACF: Residential aged care facility
UK: United Kingdom
US: United States

## References

[1] Konetzka RT, Stearns SC, Park J (2008). The staffing-outcomes relationship in nursing homes. Health Serv Res.

[2] Bostick JE, Rantz MJ, Flesner MK, Riggs CJ (2006). Systematic review of studies of staffing and quality in nursing homes. J Am Med Dir Assoc.

[3] Maas ML, Specht JP, Buckwalter KC, Gittler J, Bechen K. Nursing home staffing and training recommendations for promoting older a adults’ quality of care and life: Part 1. Deficits in the quality of care due to understaffing and undertraining. Res Gerontol Nurs. 2008;1:123–33. 10.3928/19404921-20080401-03. 10.3928/19404921-20080401-03 20078025

[4] Estabrooks C, Straus S, Flood C, Keefe J, Armstrong P, Donner G, et al. Restoring trust: COVID-19 and the future of long-term care. Ottawa, Canada: Royal Society of Canada, 2020. https://rsc-src.ca/sites/default/files/LTC%20PB%20%2B%20ES_EN_0.pdf. Accessed 23 Dec 2021.

[5] Cookson D. Mandated nursing staff to resident ratios in aged care: Summary of evidence. Wellington, New Zealand: New Zealand Nurses Organisation, 2017. https://www.nzno.org.nz/Portals/0/publications/Mandated%20nursing%20staff%20to%20resident%20ratios%20in%20aged%20care%20-%20Summary%20of%20evidence%202017.pdf?ver=sEo-tUwZBZgaa2G9irhrHg%3d%3d. Accessed 23 Dec 2021.

[6] Harrington C, Choiniere J, Goldmann M, Jacobsen FF, Lloyd L, McGregor M (2012). Nursing home staffing standards and staffing levels in six countries. J Nurs Scholarsh.

[7] Dyer SM VM, Arora N, Ross T, Winsall M, Tilden D, Crotty M. Review of international systems for long-term care of older people. Adelaide, Australia: Flinders University, 2019. https://agedcare.royalcommission.gov.au/sites/default/files/2020-09/Research%20Paper%202%20-%20Review%20of%20international%20systems%20for%20long-term%20care%20of.pdf. Accessed 23 Dec 2021.

[8] Harrington C, Schnelle JF, McGregor M, Simmons SF. The need for higher minimum staffing standards in U.S. nursing homes. Health Serv Insights. 2016;9:13–9. 10.4137/HSI.S38994. 10.4137/HSI.S38994 PMC4833431 27103819

[9] Hodgkinson B, Haesler EJ, Nay R, O'Donnell MH, McAuliffe LP. Effectiveness of staffing models in residential, subacute, extended aged care settings on patient and staff outcomes. Cochrane Database Syst Rev. 2011:Cd006563 10.1002/14651858.CD006563.pub2 10.1002/14651858.CD006563.pub2 PMC12767757 21678358

[10] Frändin K, Grönstedt H, Helbostad JL, Bergland A, Andresen M, Puggaard L (2016). Long-term effects of individually tailored physical training and activity on physical function, well-being and cognition in scandinavian nursing home residents: A randomized controlled trial. Gerontology.

[11] Speech Pathology Australia. Inquiry into caring for older Australians. Melbourne, Australia: Speech Pathology Association of Australia, 2011. https://www.pc.gov.au/inquiries/completed/aged-care/submissions/subdr752.pdf. Accessed 23 Dec 2021.

[12] Dietiticans Association of Australia. Submission from the Dietitians association of Australia to the productivity commission: caring for Older Australians Deakin, Australia2010. https://www.pc.gov.au/inquiries/completed/aged-care/submissions/sub371.pdf. Accessed 23 Dec 2021.

[13] Savvas S, Gibson S (2015). Pain management in residential aged care facilities. Aust J Gen Pract.

[14] Buchan J, Law D. A review of allied health workforce models and structures. Melboroune, Australia: department of health & human services, Victorian State Government, 2016. https://content.health.vic.gov.au/sites/default/files/migrated/files/collections/research-and-reports/r/review-of-allied-health-models-and-structures.pdf. Accessed 23 Dec 2021.

[15] Turnbull C, Grimmer-Somers K, Kumar S, May E, Law D, Ashworth E (2009). Allied, scientific and complementary health professionals: A new model for Australian allied health. Aust Health Rev.

[16] Nancarrow SA, Young G, O’Callaghan K, Jenkins M, Philip K, Barlow K. Shape of allied health: an environmental scan of 27 allied health professions in Victoria. Aust Health Rev. 2017;41:327–35. 10.1071/AH16026. 10.1071/AH16026 27509228

[17] Naccarella L (2015). Strengthening the allied health workforce: Policy, practice and research issues and opportunities. Aust Health Rev.

[18] Solomon D, Graves N, Catherwood J (2015). Allied health growth: What we do not measure we cannot manage. Hum Resour Health.

[19] Dorning H, Bardsley M. Focus on: Allied health professionals. London, UK: The Health Foundation Nuffield Trust, 2014. https://www.nuffieldtrust.org.uk/files/2018-10/1540246918_qualitywatch-focus-on-allied-health-professionals.pdf. Accessed 23 Dec 2021.

[20] Page MJ, McKenzie JE, Bossuyt PM, Boutron I, Hoffmann TC, Mulrow CD (2021). The PRISMA 2020 statement: an updated guideline for reporting systematic reviews. Syst Rev.

[21] Joanna Briggs Institute. JBI manual for evidence synthesis. Adelaide, Australia: JBI, 2020. https://synthesismanual.jbi.global. Accessed 23 Dec

[22] Ouzzani M, Hammady H, Fedorowicz Z, Elmagarmid A (2016). Rayyan—a web and mobile app for systematic reviews. Syst Rev.

[23] McHugh ML (2012). Interrater reliability: The kappa statistic. Biochem Med.

[24] Hong QN PP, Fàbregues S, Bartlett G, Boardman F, Cargo M, Dagenais P, Gagnon, M-P GF, Nicolau B, O’Cathain A, Rousseau M-C, Vedel I. Mixed Methods Appraisal Tool (MMAT), version 2018: McGill, 2018. http://mixedmethodsappraisaltoolpublic.pbworks.com/w/file/fetch/127916259/MMAT_2018_criteria-manual_2018-08-01_ENG.pdf. Accessed 23 Dec 2021.

[25] Hong QN PP, Fàbregues S, Bartlett G, Boardman F, Cargo M, Dagenais P, Gagnon, M-P GF, Nicolau B, O’Cathain A, Rousseau M-C, Vedel I. Reporting the results of the MMAT (version 2018). 2020. http://mixedmethodsappraisaltoolpublic.pbworks.com/w/file/fetch/140056890/Reporting%20the%20results%20of%20the%20MMAT.pdf. Accessed 23 Dec 2021.

[26] Tyndall J. The AACODS checklist is designed to enable evaluation and critical appraisal of grey literature. Adelaide, Australia: Flinders University, 2010. https://dspace.flinders.edu.au/xmlui/bitstream/handle/2328/3326/AACODS_Checklist.pdf. Accessed 23 Dec 2021.

[27] Bennett M, Cartwright J, Young J (2019). Is the speech-language pathology profession prepared for an ageing population? An Australian survey. Int J Speech Lang Pathol.

[28] Bern-Klug M, Kramer KW (2013). Core functions of nursing home social services departments in the United States. J Am Med Dir Assoc.

[29] Bhuyan SS, Chandak A, Gupta N, Wyant DK, Kim J, Bhatt J (2017). Provision of rehabilitation services in residential care facilities: Evidence from a national survey. Arch Phys Med Rehabil.

[30] Bonaccorsi G, Collini F, Castagnoli M, Di Bari M, Cavallini MC, Zaffarana N (2015). A cross-sectional survey to investigate the quality of care in Tuscan (Italy) nursing homes: the structural, process and outcome indicators of nutritional care. BMC Health Serv Res.

[31] Buddingh S, Liang J, Allen J, Koziak A, Buckingham J, Beaupre LA (2013). Rehabilitation for long-term care residents following hip fracture: A survey of reported rehabilitation practices and perceived barriers to delivery of care. J Geriatr Phys Ther.

[32] Burger C, Kiesswetter E, Gietl A, Pfannes U, Arens-Azevedo U, Sieber CC (2017). Size matters! Differences in nutritional care between small, medium and large nursing homes in Germany. J Nutr Health Aging.

[33] Enam N, Grampurohit N, Farber RS. Sleep management within skilled nursing facilities: A practice survey. *Occup *Ther Health Care. 2020:1–17 10.1080/07380577.2020.1846234 10.1080/07380577.2020.1846234 33228469

[34] Engh MCN, Speyer R. Management of dysphagia in nursing homes: a national survey. Dysphagia. 2021 10.1007/s00455-021-10275-7 10.1007/s00455-021-10275-7 PMC8948132 33660070

[35] Giesbrecht EM, Mortenson WB, Miller WC (2012). Prevalence and facility level correlates of need for wheelchair seating assessment among long-term care residents. Gerontology.

[36] Hirdes JP, Mitchell L, Maxwell CJ, White N. Beyond the “iron lungs of gerontology”: using evidence to shape the future of nursing homes in Canada. Can J Aging. 2011;30:371–90. 10.1017/S0714980811000304. 10.1017/S0714980811000304 21851753

[37] Hsu AT, Berta W, Coyte PC, Laporte A (2016). Staffing in Ontario’s long-term care homes: differences by profit status and chain ownership. Can J Aging.

[38] Hurley L, O’Donnell M, O’Caoimh R, Dinneen SF. Investigating the management of diabetes in nursing homes using a mixed methods approach. Diabetes Res Clin Pract. 2017;127:156–62. 10.1016/j.diabres.2017.03.010. 10.1016/j.diabres.2017.03.010 28371686

[39] Kinley J, Hockley J, Stone L, Dewey M, Hansford P, Stewart R (2014). The provision of care for residents dying in U.K. nursing care homes. Age Ageing..

[40] Koenig TL, Lee JH, Fields NL, Macmillan KR (2011). The role of the gerontological social worker in assisted living. J Gerontol Soc Work.

[41] Lee JY, Kim EY, Cho E (2011). Factors impacting the physical function of older adults in Korean long-term care hospitals. J Korean Acad Nurs.

[42] Liu X, Burns DS, Hilliard RE, Stump TE, Unroe KT (2015). Music therapy clinical practice in hospice: differences between home and nursing home delivery. J Music Ther.

[43] Livingstone I, Hefele J, Leland N (2021). Characteristics of nursing home providers with distinct patterns of physical and occupational therapy staffing. J Appl Gerontol.

[44] Livingstone I, Hefele J, Leland N. Physical and occupational therapy staffing patterns in nursing homes and their association with long-stay resident outcomes and quality of care. J Aging Soc Policy. 2020:1–19 10.1080/08959420.2020.1824544 10.1080/08959420.2020.1824544 33016241

[45] Livingstone I, Hefele J, Nadash P, Barch D, Leland N. The relationship between quality of care, physical therapy, and occupational therapy staffing levels in nursing homes in 4 Years’ follow-up. J Am Med Dir Assoc. 2019;20:462–9. 10.1016/j.jamda.2019.02.002. 10.1016/j.jamda.2019.02.002 30954134

[46] McArthur C, Hirdes J, Berg K, Giangregorio L (2015). Who receives rehabilitation in canadian long-term care facilities? A cross-sectional study. Physiother Can.

[47] McArthur C, Hirdes J, Chaurasia A, Berg K, Giangregorio L. Quality changes after implementation of an episode of care model with strict criteria for physical therapy in Ontario’s long-term care homes. Health Serv Res. 2018;53:4863–85. 10.1111/1475-6773.13020. 10.1111/1475-6773.13020 PMC6232515 30091461

[48] Roberts AR, Bowblis JR (2018). How does rurality influence the staffing of social service departments in nursing homes?. Gerontologist.

[49] Roberts AR, Bowblis JR (2017). Who hires social workers? Structural and contextual determinants of social service staffing in nursing homes. Health Soc Work.

[50] SkinnarsJosefsson M, Nydahl M, Persson I, Mattsson SY (2017). Quality indicators of nutritional care practice in elderly care. J Nutr Health Aging.

[51] Stargatt J, Bhar SS, Davison TE, Pachana NA, Mitchell L, Koder D (2017). The availability of psychological services for aged care residents in Australia: a survey of facility staff. Aust Psychol.

[52] Sterke S, Nascimento da Cunha AP, Oomen H, Voogt L, Goumans M. Physiotherapy in nursing homes. A qualitative study of physiotherapists' views and experiences. BMC Geriatr. 2021;21:150 10.1186/s12877-021-02080-6 10.1186/s12877-021-02080-6 PMC7923506 33648440

[53] Temkin-Greener H, Lee T, Caprio T, Cai S (2019). Rehabilitation therapy for nursing home residents at the end-of-life. J Am Med Dir Assoc.

[54] Tyler DA, Feng Z, Leland NE, Gozalo P, Intrator O, Mor V (2013). Trends in postacute care and staffing in US nursing homes, 2001–2010. J Am Med Dir Assoc.

[55] van Nie-Visser NC, Meijers JM, Schols JM, Lohrmann C, Bartholomeyczik S, Halfens RJ (2011). Comparing quality of nutritional care in Dutch and German nursing homes. J Clin Nurs.

[56] StewartBrown. 2020 Allied health deep dive survey. Sydney, Australia: StewartBrown, 2020. https://www.stewartbrown.com.au/images/documents/StewartBrown_Allied_Health_Deep_Dive_Survey__Feb_2021.pdf. Accessed 23 Dec 2021.

[57] StewartBrown. Aged care financial performance survey: Residential care report - June 2017. Sydney, Australia: StewartBrown, 2017. https://www.stewartbrown.com.au/images/documents/StewartBrown_-_ACFPS_Residential_Care_Report_June-2017.pdf. Accessed 23 Dec 2021.

[58] StewartBrown. Aged care financial performance survey: Residential care report - December 2017. Sydney, Australia: StewartBrown, 2017. https://www.stewartbrown.com.au/images/documents/StewartBrown---ACFPS-Residential-Care-Report-December-2017.pdf. Accessed 23 Dec 2021.

[59] StewartBrown. Aged care financial performance survey: Residential care report - September 2017. Sydney, Australia: StewartBrown, 2017. https://www.stewartbrown.com.au/images/documents/StewartBrown---ACFPS-Residential-Care-Report-Sept-2017.pdf. Accessed 23 Dec 2021.

[60] StewartBrown. Aged care financial performance survey: Residential care repot - March 2018. Sydney, Australia: StewartBrown, 2018. https://www.stewartbrown.com.au/images/documents/StewartBrown---ACFPS-Residential-Care-Report-March-2018.pdf. Accessed 23 Dec 2021.

[61] StewartBrown. Aged Care Financial Performance Survey: Sector report (2018 financial year). Sydney, Australia: StewartBrown, 2018. https://www.stewartbrown.com.au/images/documents/StewartBrown---ACFPS-Sector-Report-June-2018.pdf. Accessed 23 Dec 2021.

[62] StewartBrown. Aged Care Financial Performance Survey: Sector report (six months ended December 2018). Sydney, Australia: StewartBrown, 2018. https://www.stewartbrown.com.au/images/documents/StewartBrown---ACFPS-Sector-Financial-Performance-Report-December-2018.pdf. Accessed 23 Dec 2021.

[63] StewartBrown. Aged care financial performance survey: Sector report (nine months ended March 2019). Sydney, Australia: StewartBrown, 2019. https://www.stewartbrown.com.au/images/documents/StewartBrown---ACFPS-Sector-Financial-Performance-Report-March-2019.pdf. Accessed 23 Dec 2021.

[64] StewartBrown. Aged care financial performance survey: Aged care sector report. Sydney, Australia: StewartBrown, 2019. https://www.stewartbrown.com.au/images/documents/StewartBrown---FY19-Aged-Care-Financial-Performance-Survey-Report.pdf. Accessed 23 Dec 2021.

[65] StewartBrown. Aged care financial performance survey: Aged care sector report (for six months end 31 December 2019). Sydney, Australia: StewartBrown, 2019. https://www.stewartbrown.com.au/images/documents/StewartBrown---Aged-Care-Financial-Performance-Survey-Sector-Report-December-2019.pdf. Accessed 23 Dec 2021.

[66] StewartBrown. Aged care Financial performance survey: Sector report (nine months ended 31 March 2020). Sydney, Australia: StewartBrown, 2020. https://www.stewartbrown.com.au/images/documents/StewartBrown_-_Aged_Care_Financial_Performance_Survey_Sector_March_2020.pdf. Accessed 23 Dec 2021.

[67] StewartBrown. Aged care financial performance survey: Aged care sector report (for the year ended 30 June 2020). Sydney, Australia: StewartBrown, 2020. https://www.stewartbrown.com.au/images/documents/StewartBrown_-_Aged_Care_Financial_Performance_Survey_Sector_Report_June_2020.pdf. Accessed 23 Dec 2021.

[68] StewartBrown. Aged care financial performance survey: Aged care sector report (three month ended 30 September 2020). Sydney, Australia: StewartBrown, 2020. https://www.stewartbrown.com.au/images/documents/StewartBrown_-_ACFPS_Sector_Financial_Performance_Report_September_2020.pdf. Accessed 23 Dec 2021.

[69] StewartBrown. Aged care sector report: For the six months ended 31 December 2020. Sydney, Australia: StewartBrown, 2020. https://www.stewartbrown.com.au/images/documents/StewartBrown_-_ACFPS_Financial_Performance_Sector_Report_December_2020.pdf. Accessed 23 Dec 2021.

[70] Mavromaras K, Knight G, Isherwood L, Crettenden A, Flavel J, Karmel T, et al. The aged care workforce. Canberra, Australia: Department of Health, Australian Government, 2017. https://gen-agedcaredata.gov.au/www_aihwgen/media/Workforce/The-Aged-Care-Workforce-2016.pdf. Accessed 23 Dec 2021.

[71] King D, Mavromaras K, Wei Z, He B, Healy J, Macaitis K, et al. The aged care workforce 2012: Department of Health, Australian Government, 2013. http://www.agedcarecrisis.com/images/pdf/The_Aged_Care_Workforce_Report.pdf. Accessed 23 Dec 2021.

[72] Department of Health. 2020 Aged care workforce census report. Canberra, Australia: Australian Government, 2020. https://www.health.gov.au/sites/default/files/documents/2021/10/2020-aged-care-workforce-census.pdf. Accessed 23 Dec 2021.

[73] Eagar K, Westera A, Snoek M, Kobel C, Loggie C, Gordon R. How Australian residential aged care staffing levels compare with international and national benchmarks. Wollongong, Australia: Centre for Health Service Development, Australian Health Services Research Institute, 2019. https://agedcare.royalcommission.gov.au/sites/default/files/2019-12/research-paper-1.pdf. Accessed 23 Dec 2021.

[74] de Souto Barreto P, Morley J, Chodzko-Zajko W, H. Pitkala K, Weening-Djiksterhuis E, Rodriguez-Mañas L, et al. Recommendations on physical activity and exercise for older adults living in long-term care facilities: A taskforce report. J Am Med Dir Assoc. 2016;17:381–92 10.1016/j.jamda.2016.01.021 10.1016/j.jamda.2016.01.021 27012368

[75] Ontario Ministry of Health. A better place to live, a better place to work: Ontario Ministry of Health, 2020. https://files.ontario.ca/mltc-ontario-long-term-care-staffing-plan-2021-2025-en-2020-12-17.pdf. Accessed 23 Dec 2021.

[76] Nancarrow S. Why is it so difficult to understand and plan the allied health workforce in Australia – and what is the solution? Australia: AHP Workforce, 2021. https://ahpworkforce.com/challenges-understanding-and-planning-the-allied-health-workforce-in-australia/. Accessed 23 Dec 2021.

