# Supplementary material for: Therapy-based allied health delivery in residential aged care, trends, factors, and outcomes: a systematic review
Source: BMC Geriatr. 2022 Aug 28;22:712. doi: 10.1186/s12877-022-03386-9 (PMC9420184; doi:10.1186/s12877-022-03386-9)
Supplement: Supplementary file 1 — Additional file 1: Medline OVID search strategy. [file 12877_2022_3386_MOESM1_ESM.docx]

# Appendix 1

**Table 1**. Medline Ovid Search strategy

| 1 | exp Allied Health Personnel/ or Audiologists/ or Case Managers/ or Nutritionists/ or Occupational Therapists/ or Pharmacists/ or Physical Therapists/ or exp Allied Health Occupations/ or Chiropractic/ or Orthoptics/ or Pharmacy/ or Podiatry/ | 137507 |
| --- | --- | --- |
| 2 | Allied health*.ti,ab. | 9966 |
| 3 | (Physiotherap* or physical therap*).ti,ab. | 50754 |
| 4 | (occupational therap* or speech therap* or speech patholog* or speech-language therap* or case manager* or case worker* or dietician* or nutrition* or pharmac* or mental health work* or mental health practition* or psycholog* or clinical psycholog* or social work* or sociolog* or podiat* or chiropractor* or music therap* or counsell* or exercise physiolog* or orthot* or prosthetic* or radiograph* or radiation therap* or diversional therap* or orthopt* or exercise therap* or therap*).ti,ab. | 1818797 |
| 5 | or/1-4 | 1960240 |
| 6 | nursing homes/ or intermediate care facilities/ or skilled nursing facilities/ or homes for the aged/ or assisted living facilities/ | 47414 |
| 7 | (aged care or assisted living facilt* or assisted living or homes for the aged or nursing home* or skilled nursing facilit* or senior care).ti,ab. | 39320 |
| 8 | (residential adj (aged or elderly or geriatric)).ti,ab. | 1345 |
| 9 | Long Term Care/ or (residential facilit* or residential care).ti,ab. | 30976 |
| 10 | ((long term or extended) adj care).ti,ab. | 22699 |
| 11 | or/9-10 | 43466 |
| 12 | exp Aged/ or (aged or elder* or old*).ti,ab. | 4805124 |
| 13 | and/11-12 | 23088 |
| 14 | or/6-8,13 | 77678 |
| 15 | ((Care or service or treatment or activit* or group or class or therapy or staff* or workforce).ti,ab. | 529511 |
| 16 | Health Resources/ or Health Services Accessibility/ or Medically Underserved Area/ or Quality Indicators, Health Care/ or "Standard of Care"/ | 117183 |
| 17 | 15 or 16 | 618719 |
| 18 | 5 and 14 and 17 | 1260 |
| 19 | limit 18 to dt=20110601-20210716 | 711 |
| 20 | limit 19 to english language | 681 |

Note: The decision to limit the review to therapy-based allied health was made after finding the results of all allied health professions were too diverse to synthesise. Therefore, search results were manually narrowed during screening to remove non-therapy-based allied health professions (i.e., pharmacy and audiology).
