# Supplementary material for: Therapy-based allied health delivery in residential aged care, trends, factors, and outcomes: a systematic review
Source: BMC Geriatr. 2022 Aug 28;22:712. doi: 10.1186/s12877-022-03386-9 (PMC9420184; doi:10.1186/s12877-022-03386-9)
Supplement: Supplementary file 2 — Additional file 2: Websites searched in grey literature search strategy. [file 12877_2022_3386_MOESM2_ESM.docx]

# Appendix 2

**Table 1.** Websites searched in grey literature search strategy

| Academy of Nutrition and Dietetics |
| --- |
| American Podiatric Medical Association (apma) |
| Age UK |
| Aged and Community Services Australia (ACSA) |
| Agency for Healthcare Research and Quality (AHRQ) |
| AHP Workforce |
| Allied Health Aotearoa New Zealand (AHANZ) |
| Association of Allied health Professional (AAHP) |
| Allied Health Professions Australia (AHPA) |
| Allied Health Professions Federation (AHFP) |
| American Music Therapy Association (AMTA) |
| American Occupational Therapy Association (AOTA) |
| American Physical Therapy Association (APTA) |
| American Speech Language Hearing Association (ASHA) |
| American Therapeutic Recreation Association (ATRA) |
| Anglicare Australia |
| Association of Independent Retirees |
| Audiology Australia |
| Australia and New Zealand Society for Geriatric Medicine (ANZGM) |
| Australian Association of Gerontology (AAG) |
| Australian Association of Social Workers (AASW) |
| Australian Community Industry Alliance (ACIA) |
| Australian Healthcare and Hospitals Association (AHHA) |
| Australian Institute of Health and Welfare (AIHW) |
| Australian Physiotherapy Association (APA) |
| Australian Primary Health Care Nurses Association (APNA) |
| Australian Psychological Society (APS) |
| Australasian Services Care Network (ASCN) |
| Baptist Care Australia |
| British Association for Music Therapy (BAMT) |
| British Columbia Ministry of Health |
| Canadian Association of Music Therapists (CAMT) |
| Canadian Association of Occupational Therapists (CAOT) |
| Canadian Association of Social Workers (CASW) |
| Canadian Government |
| Canadian Institute for Health Information (CIHI) |
| Canadian Institutes of Health Research (CIHR) |
| Canadian Physiotherapy Association (APA) |
| Canadian Psychological Association (CPA) |
| Canadian Therapeutic Recreation Association (CTRA) |
| Carers Australia |
| Catholic Health Australia |
| Centre for Health and the Public interest (CHPI) |
| Centre for Workforce Intelligence (CfWI) |
| Centres for Medicare and Medicaid Services (CMS) |
| Chartered Society of Physiotherapy (CSP) |
| Commonwealth Fund |
| COTA Australia |
| Deloitte |
| Australian Government Department of Health |
| Dieticians’ Australia |
| Diversional & Recreation Therapy Australia (DRTA) |
| European Institute of Innovation and Technology (EIT) |
| Exercise & Sports Science Australia (ESSA) |
| Federation of Ethnic Communities' Councils of Australia (FECCA) |
| Google Advanced |
| Government of Alberta |
| Government of Western Australia, Department of Health |
| Health Foundation |
| Health Resources and Services Administration (HRSA) |
| Kings Fund |
| KPMG |
| Leading Age Services Australia (LASA) |
| Legacy Australia |
| LGBTQ+ Health Alliance |
| Meaningful Ageing Australia |
| Ministry of Health New Zealand |
| National Association of Social Workers (NASW) |
| National Rural Health Alliance (NRHA) |
| New Zealand Work Research Institute (NZWRI) |
| National Health Service (NHS) |
| NHS confederation |
| Nuffield Trust |
| Occupational Therapy Australia (OTA) |
| Organisation for Economic Co-operation and Development (OECD) |
| Older Persons Advocacy Network (OPAN) |
| Palliative Care Australia |
| Physiotherapy Board, New Zealand |
| Physiotherapy New Zealand (PNZ) |
| Partners in Culturally Appropriate Care (PICAC) Alliance |
| PricewaterhouseCoopers (PwC) |
| Province of Manitoba |
| RAND corporation |
| The Returned & Services League of Australia |
| Royal College of Occupational Therapists (RCOT) |
| Royal College of Speech and Language Therapists (RCSLT) |
| Royal Society of Canada (RSC) |
| Saskatchewan Health Authority |
| Services for Australian Rural and Remote Allied Health (SARRAH) |
| Skills for Care |
| New Zealand Speech-language Therapists' Association (NZSTA) |
| Speech-Language & Audiology Canada (SAC) |
| StewartBrown |
| British Psychological Society (BPS) |
| National Academy of Medicine (NAM) |
| US Department of Health and Human Services (HHS) |
| Victorian Government Department of Health (DH) |
| World Economic Forum (WEF) |
| World Health Organization (WHO) |

Note: more than one search was conducted in many of these websites to allow for the use of synonyms in searching as Boolean operators could not be used for all searches.
