# Supplementary material for: Therapy-based allied health delivery in residential aged care, trends, factors, and outcomes: a systematic review
Source: BMC Geriatr. 2022 Aug 28;22:712. doi: 10.1186/s12877-022-03386-9 (PMC9420184; doi:10.1186/s12877-022-03386-9)
Supplement: Supplementary file 3 — Additional file 3: MMAT scores. [file 12877_2022_3386_MOESM3_ESM.docx]

# Appendix 3

**Table 1:** MMAT scores

| **Peer-reviewed article** | **1.1** | **1.2** | **1.3** | **1.4** | **1.5** | **3.1** | **3.2** | **3.3** | **3.4** | **3.5** | **4.1** | **4.2** | **4.3** | **4.4** | **4.5** | **5.1** | **5.2** | **5.3** | **5.4** | **5.5** | **Score** |
| --- | --- | --- | --- | --- | --- | --- | --- | --- | --- | --- | --- | --- | --- | --- | --- | --- | --- | --- | --- | --- | --- |
| Bennet (2019)[27] |  |  |  |  |  |  |  |  |  |  | ● | ● | ● | ● | ● |  |  |  |  |  | 80% |
| Bern-Klug (2013)[28] |  |  |  |  |  |  |  |  |  |  | ● | ● | ● | ● | ● |  |  |  |  |  | 60% |
| Bonaccorsi (2015)[30] |  |  |  |  |  | ● | ● | ● | ● | ● |  |  |  |  |  |  |  |  |  |  | 80% |
| Buddingh (2013)[31] |  |  |  |  |  |  |  |  |  |  | ● | ● | ● | ● | ● |  |  |  |  |  | 60% |
| Burger (2017)[32] |  |  |  |  |  |  |  |  |  |  | ● | ● | ● | ● | ● |  |  |  |  |  | 100% |
| Enam (2013)[33] |  |  |  |  |  |  |  |  |  |  | ● | ● | ● | ● | ● |  |  |  |  |  | 60% |
| Engh (2021)[34] |  |  |  |  |  |  |  |  |  |  | ● | ● | ● | ● | ● |  |  |  |  |  | 80% |
| Giesbracht (2012)[35] |  |  |  |  |  |  |  |  |  |  | ● | ● | ● | ● | ● |  |  |  |  |  | 60% |
| Hirdes (2011)[36] |  |  |  |  |  |  |  |  |  |  | ● | ● | ● | ● | ● |  |  |  |  |  | 100% |
| Hurley (2017)[38] |  |  |  |  |  |  |  |  |  |  |  |  |  |  |  | ● | ● | ● | ● | ● | 80% |
| Kinley (2014)[39] |  |  |  |  |  |  |  |  |  |  | ● | ● | ● | ● | ● |  |  |  |  |  | 80% |
| Koenig (2011)[40] | ● | ● | ● | ● | ● |  |  |  |  |  |  |  |  |  |  |  |  |  |  |  | 100% |
| Lee (2011)[41] |  |  |  |  |  | ● | ● | ● | ● | ● |  |  |  |  |  |  |  |  |  |  | 100% |
| Liu (2015)[42] |  |  |  |  |  | ● | ● | ● | ● | ● |  |  |  |  |  |  |  |  |  |  | 100% |
| Livingstone (2019)[45] |  |  |  |  |  | ● | ● | ● | ● | ● |  |  |  |  |  |  |  |  |  |  | 100% |
| Livingstone (2020)[44] |  |  |  |  |  | ● | ● | ● | ● | ● |  |  |  |  |  |  |  |  |  |  | 100% |
| Livingstone (2021)[43] |  |  |  |  |  | ● | ● | ● | ● | ● |  |  |  |  |  |  |  |  |  |  | 100% |
| McArthur (2015)[46] |  |  |  |  |  | ● | ● | ● | ● | ● |  |  |  |  |  |  |  |  |  |  | 100% |
| McArthur (2018)[47] |  |  |  |  |  | ● | ● | ● | ● | ● |  |  |  |  |  |  |  |  |  |  | 100% |
| Roberts (2017)[49] |  |  |  |  |  | ● | ● | ● | ● | ● |  |  |  |  |  |  |  |  |  |  | 100% |
| Roberts (2018)[48] |  |  |  |  |  | ● | ● | ● | ● | ● |  |  |  |  |  |  |  |  |  |  | 100% |
| Skinnars (2017)[50] |  |  |  |  |  | ● | ● | ● | ● | ● |  |  |  |  |  |  |  |  |  |  | 80% |
| Stargatt (2017)[51] |  |  |  |  |  |  |  |  |  |  | ● | ● | ● | ● | ● |  |  |  |  |  | 40% |
| Sterke (2017)[52] | ● | ● | ● | ● | ● |  |  |  |  |  |  |  |  |  |  |  |  |  |  |  | 60% |
| Temkin-Greener (2019)[53] |  |  |  |  |  | ● | ● | ● | ● | ● |  |  |  |  |  |  |  |  |  |  | 100% |
| Tyler (2019)[54] |  |  |  |  |  |  |  |  |  |  | ● | ● | ● | ● | ● |  |  |  |  |  | 80% |
| Van Nie-Visser (2011)[55] |  |  |  |  |  | ● | ● | ● | ● | ● |  |  |  |  |  |  |  |  |  |  | 80% |

● Yes, ● No, ● Can’t tell/unknown.

**Table 2:** ACCODS scores

| **Grey literature article** | **Authority** | **Accuracy** | **Coverage** | **Objectivity** | **Date** | **Significance** | **Summary score** |
| --- | --- | --- | --- | --- | --- | --- | --- |
| Department of Health, Australia (2013)[71] | ● | ● | ● | ● | ● | ● | 100% |
| Department of Health, Australia (2017)[70] | ● | ● | ● | ● | ● | ● | 100% |
| Department of Health, Australia (2020)[72] | ● | ● | ● | ● | ● | ● | 100% |
| StewartBrown (2021)[56] | ● | ● | ● | ● | ● | ● | 66.7% |
| StewartBrown (2020)[69] | ● | ● | ● | ● | ● | ● | 66.7% |
| StewartBrown (2020)[68] | ● | ● | ● | ● | ● | ● | 66.7% |
| StewartBrown (2020)[67] | ● | ● | ● | ● | ● | ● | 66.7% |
| StewartBrown (2020)[66] | ● | ● | ● | ● | ● | ● | 66.7% |
| StewartBrown (2019)[65] | ● | ● | ● | ● | ● | ● | 66.7% |
| StewartBrown (2019)[64] | ● | ● | ● | ● | ● | ● | 66.7% |
| StewartBrown (2019)[63] | ● | ● | ● | ● | ● | ● | 66.7% |
| StewartBrown (2018)[62] | ● | ● | ● | ● | ● | ● | 66.7% |
| StewartBrown (2018)[61] | ● | ● | ● | ● | ● | ● | 66.7% |
| StewartBrown (2018)[60] | ● | ● | ● | ● | ● | ● | 66.7% |
| StewartBrown (2017)[59] | ● | ● | ● | ● | ● | ● | 66.7% |
| StewartBrown (2017)[58] | ● | ● | ● | ● | ● | ● | 66.7% |
| StewartBrown (2017)[57] | ● | ● | ● | ● | ● | ● | 66.7% |

● Yes, ● No, ● Can’t tell/unknown
