# Supplementary material for: Therapy-based allied health delivery in residential aged care, trends, factors, and outcomes: a systematic review
Source: BMC Geriatr. 2022 Aug 28;22:712. doi: 10.1186/s12877-022-03386-9 (PMC9420184; doi:10.1186/s12877-022-03386-9)
Supplement: Supplementary file 4 — Additional file 4: Factors associated with allied health service delivery. [file 12877_2022_3386_MOESM4_ESM.docx]

# Appendix 4

**Table 1**. Factors associated with allied health service delivery

| **Summary** | **Country** | **Effect size** | | | | | | **Measure** |
| --- | --- | --- | --- | --- | --- | --- | --- | --- |
| **System level** | | | | | | | | |
| ***Funding*** | | | | | | | | |
| Change in proportion of residents receiving occupational services pre/post policy change[47] | Canada | Pre: 2.5^a^ (-) | | | | | | Percentage |
|  |  | Post: 1.0^a^ (-) | | | | | |  |
| Change in proportion of residents receiving any amount physiotherapy service pre/post policy change[47] | Canada | Pre: 84.6^a^ (-) | | | | | | Percentage |
|  |  | Post: 56.6^a^ (-) | | | | | |  |
| Change in proportion of residents receiving speech pathology services pre/post policy change[47] | Canada | Pre: 0.3 | | | | | | Percentage |
|  |  | Post: 0.3 | | | | | |  |
| ***State/Province*** | | | | | | | | |
| Likelihood of receiving any physiotherapy (refence group British Columbia)[46] | Canada | Manitoba: 0.42** (0.39, 0.45) | | | | | | Adjusted odds ratio |
|  |  | Newfoundland: 2.50** (2.22, 2.82) | | | | | |  |
|  |  | Nova Scotia: 3.01** (2.77, 3.41) | | | | | |  |
|  |  | Saskatchewan: 0.81** (0.76, 0.86) | | | | | |  |
|  |  | Yukon: 2.45** (1.89, 3.19) | | | | | |  |
| Percentage of residents receiving physiotherapy services by US Centres for Medicare and Medicare Services region categories[43] | US |  | High staffing levels | Medium staffing levels | | | Low staffing levels | Percent |
|  |  | Region 1 | 37.9* | 38.6* | | | 23.5* |  |
|  |  | Region 2 | 48.7* | 39.4* | | | 11.9* |  |
|  |  | Region 3 | 42.3* | 39.4* | | | 18.4* |  |
|  |  | Region 4 | 35.9* | 36.9* | | | 27.2* |  |
|  |  | Region 5 | 29.8* | 32.1* | | | 38.2* |  |
|  |  | Region 6 | 19.7* | 32.5* | | | 47.8* |  |
|  |  | Region 7 | 15.4* | 25.8* | | | 58.7* |  |
|  |  | Region 8 | 31.5* | 24.7* | | | 43.8* |  |
|  |  | Region 9 | 54.9* | 24.3* | | | 20.8* |  |
|  |  | Region 10 | 62.6* | 25.3* | | | 12.1* |  |
| Percentage of residents receiving physiotherapy assistant services by US Centres for Medicare and Medicare Services region categories[43] | US |  | High staffing levels | Medium staffing levels | | | Low staffing levels | Percent |
|  |  | Region 1 | 25.6* | 36.8* | | | 37.6* |  |
|  |  | Region 2 | 22.1* | 33.7* | | | 44.2* |  |
|  |  | Region 3 | 35.1* | 39.4* | | | 25.5* |  |
|  |  | Region 4 | 42.2* | 33.2* | | | 24.6* |  |
|  |  | Region 5 | 41.6* | 32.1* | | | 26.4* |  |
|  |  | Region 6 | 26.6* | 33.4* | | | 40.0* |  |
|  |  | Region 7 | 19.8* | 36.1* | | | 44.2* |  |
|  |  | Region 8 | 19.7* | 22.8* | | | 57.5* |  |
|  |  | Region 9 | 32.8* | 26.2* | | | 41.1* |  |
|  |  | Region 10 | 47.2* | 25.4* | | | 27.4* |  |
| Percentage of residents receiving physiotherapy aide services by US Centres for Medicare and Medicare Services region categories[43] | US |  | Physiotherapy aides | No physiotherapy aides | | | | Percent |
|  |  | Region 1 | 29.8* | 70.2* | | | |  |
|  |  | Region 2 | 47.7* | 52.3* | | | |  |
|  |  | Region 3 | 38.9* | 61.1* | | | |  |
|  |  | Region 4 | 34.0* | 66.0* | | | |  |
|  |  | Region 5 | 27.8* | 72.2* | | | |  |
|  |  | Region 6 | 28.7* | 71.3* | | | |  |
|  |  | Region 7 | 17.2* | 82.8* | | | |  |
|  |  | Region 8 | 28.7* | 71.3* | | | |  |
|  |  | Region 9 | 34.8* | 65.2* | | | |  |
|  |  | Region 10 | 31.7* | 68.3* | | | |  |
| Percentage of residents receiving occupational therapy services by US Centres for Medicare and Medicare Services region categories[43] | US |  | High staffing levels | Medium staffing levels | | | Low staffing levels | Percent |
|  |  | Region 1 | 43.5* | 36.2* | | | 20.3* |  |
|  |  | Region 2 | 39.4* | 38.5* | | | 22.2* |  |
|  |  | Region 3 | 42.2* | 38.5* | | | 19.4* |  |
|  |  | Region 4 | 36.4* | 39.2* | | | 24.5* |  |
|  |  | Region 5 | 31.6* | 31.7* | | | 36.7* |  |
|  |  | Region 6 | 19.8* | 32.0* | | | 48.3* |  |
|  |  | Region 7 | 17.0* | 27.5* | | | 55.5* |  |
|  |  | Region 8 | 28.7* | 21.7* | | | 49.6* |  |
|  |  | Region 9 | 52.7* | 25.6* | | | 21.7* |  |
|  |  | Region 10 | 60.2* | 23.7* | | | 16.1* |  |
| Percentage of residents receiving occupational therapy assistant services by US Centres for Medicare and Medicare Services region categories[43] | US |  | High staffing levels | Medium staffing levels | | Low staffing levels | | Percent |
|  |  | Region 1 | 25.5* | 37.3* | | 37.2* | |  |
|  |  | Region 2 | 20.7* | 32.8* | | 46.5* | |  |
|  |  | Region 3 | 36.0* | 39.4* | | 24.6* | |  |
|  |  | Region 4 | 42.9* | 34.4* | | 22.6* | |  |
|  |  | Region 5 | 40.5* | 32.9* | | 26.6* | |  |
|  |  | Region 6 | 32.1* | 35.2* | | 32.7* | |  |
|  |  | Region 7 | 17.9* | 30.7* | | 51.5* | |  |
|  |  | Region 8 | 17.5* | 21.9* | | 60.6* | |  |
|  |  | Region 9 | 27.4* | 25.2* | | 47.5* | |  |
|  |  | Region 10 | 42.2* | 26.2* | | 31.6* | |  |
| Percentage of residents receiving occupational therapy aide services by US Centres for Medicare and Medicare Services region categories[43] | US |  | Occupational therapy aides | | No occupational therapy aides | | | Percent |
|  |  | Region 1 | 8.6* | | 91.5* | | |  |
|  |  | Region 2 | 18.0* | | 82.0* | | |  |
|  |  | Region 3 | 11.5* | | 88.6* | | |  |
|  |  | Region 4 | 11.6* | | 88.4* | | |  |
|  |  | Region 5 | 10.7* | | 89.3* | | |  |
|  |  | Region 6 | 13.7* | | 86.3* | | |  |
|  |  | Region 7 | 7.2* | | 92.8* | | |  |
|  |  | Region 8 | 11.7* | | 88.3* | | |  |
|  |  | Region 9 | 15.1* | | 84.9* | | |  |
|  |  | Region 10 | 8.3* | | 91.7* | | |  |
| ***Rurality*** | | | | | | | | |
| Influence of rurality on proportion of department staffed by qualified social workers (reference group urban RACFs)[48] | US | Micropolitan city: -4.5** (0.850) (-) | | | | | | Percent (standard error) |
|  |  | Small rural town: -5.46** (1.070) (-) | | | | | |  |
|  |  | Isolated small town: -7.46** (1.329) (-) | | | | | |  |
| Influence of rurality on total social service staff hours per resident per day (reference group urban RACFs)[48] | US | Micropolitan city: -0.009** (0.002) (-) | | | | | | Coefficient estimates (standard error) |
|  |  | Small rural town: -0.017** (0.003) (-) | | | | | |  |
|  |  | Isolated small town: -0.020** (0.004) (-) | | | | | |  |
| Probability of urban residents’ rehabilitative therapy in last month of life[53] | US | Low/medium therapy: 1.559** (0.219) (-) | | | | | | Relative risk ratio (standard error) |
|  |  | High/ultrahigh therapy: 1.879 (0.062) | | | | | |  |
| **Facility level factors** | | | | | | | | |
| **Facility size** | | | | | | | | |
| Likelihood of physiotherapy services in facility by facility size (reference group small)[29] | US | Medium (11–25 beds): 1.23 (0.930, 1.627) | | | | | | Odds ratio (95% confident interval) |
|  |  | Large (26–100 beds): 1.86** (1.361, 2.540) (+) | | | | | |  |
|  |  | Extra-large (≥100 beds): 2.48** (1.614, 3.803) (+) | | | | | |  |
| Likelihood of occupational therapy services in facility by facility size (reference group small)[29] | US | Medium (11–25 beds): 1.25 (0.940, 1.668) | | | | | | Odds ratio (95% confident interval) |
|  |  | Large (26–100 beds): 1.97** (1.428, 2.713) (+) | | | | | |  |
|  |  | Extra Large (≥100 beds): 2.55**(1.655, 3.938) (+) | | | | | |  |
| Hours of social service staffing by facility size^[a], [48]^ | US | Small: 0.15–0.25^a^ (-) | | | | | | Hours per resident per day |
|  |  | >100 beds: 0.08–0.12^a^ | | | | | |  |
| Percentage point change in use of qualified social service worker[49] | US | Large size >121 beds: 0.175** (0.028) (+) | | | | | | Coefficient estimates (standard error) |
|  |  | Size (# beds by 10s): 0.009**(0.002) (+) | | | | | |  |
|  |  | Interaction (# beds by 10s x >121+ beds): −0.009**(0.002) | | | | | |  |
| Mean hours per resident per day of physiotherapy staffing by facility size[43-45] | US | Small (6–81 beds): 0.11** (-) | | | | | | Mean hours per resident per day |
|  |  | Medium (82–119 beds): 0.09** | | | | | |  |
|  |  | Large (120–816 beds): 0.10** | | | | | |  |
| Mean hours per resident per day of occupational therapy staffing by facility size[43-45] | US | Small (6–81 beds): 0.10** (-) | | | | | | Mean hours per resident per day |
|  |  | Medium (82–119 beds): 0.08** | | | | | |  |
|  |  | Large (120–816 beds): 0.09** | | | | | |  |
| Mean hours per resident per day of physiotherapy assistant staffing by facility size[44] | US | Small (6–81 beds): 0.12** (-) | | | | | | Mean hours per resident per day |
|  |  | Medium (82–119 beds): 0.11** | | | | | |  |
|  |  | Large (120–816 beds): 0.11** | | | | | |  |
| Mean hours per resident per day of physiotherapy aide staffing by facility size[44] | US | Small (6–81 beds): 0.02** (-) | | | | | | Mean hours per resident per day |
|  |  | Medium (82–119 beds): 0.02** | | | | | |  |
|  |  | Large (120–816 beds): 0.02** | | | | | |  |
| Mean hours per resident per day of occupational therapy assistant staffing by facility size[44] | US | Small (6–81 beds): 0.09 | | | | | | Mean hours per resident per day |
|  |  | Medium (82–119 beds): 0.09 | | | | | |  |
|  |  | Large (120–816 beds): 0.09 | | | | | |  |
| Mean hours per resident per day of occupational therapy aide staffing by facility size[44] | US | Small (6–81 beds): 0.01 | | | | | | Mean hours per resident per day |
|  |  | Medium (82–119 beds): 0.01 | | | | | |  |
|  |  | Large (120–816 beds): 0.01 | | | | | |  |
| ***Ownership*** | | | | | | | | |
| Likelihood of physiotherapy services in for profit RACFs (reference group not for profit)[29] | US | 0.704** (0.554, 0.895) (+) | | | | | | Odds ratio (95% confidence interval) |
| Likelihood of occupational therapy services in for profit RACFs (reference group not for profit)[29] | US | 0.599** (0.470, 0.765) (+) | | | | | | Odds ratio (95% confidence interval) |
| Probability of residents receiving rehabilitative therapy at end- of-life in for profit facilities[53] | US | Low/medium therapy:^b^ 1.182** (0.048) (+) | | | | | | Relative risk ratio (standard error) |
|  |  | High/ultrahigh therapy:^b^ 2.126** (0.091) (+) | | | | | |  |
| Percentage point change in use of qualified social service worker by profit status (reference group for-profit)[49] | US | Non-profit: 0.016 (0.010) | | | | | | Coefficient estimates (standard error) |
| Percentage point change in use of paraprofessionals to deliver social services by profit status (reference group for-profit)[49] | US | Non-profit: -0.046* (0.011) (-) | | | | | | Coefficient estimates (standard error) |
| Percentage point change in use of interprofessional teams to deliver social services by profit status (reference group for-profit)[49] | US | Non-profit: -0.027** (0.013) (-) | | | | | | Coefficient estimates (standard error) |
| Minutes of difference in therapy provides in independent not for profit facilities compared to for profit chain facilities[37] | Canada | 2.1** (+) | | | | | | Minutes |
| Mean hours per resident per day of physiotherapy staffing by for profit status[44] | US | For profit: 0.09** | | | | | | Mean hours per resident per day |
|  |  | Non-profit: 0.11** (+) | | | | | |  |
| Mean hours per resident per day of physiotherapy assistant staffing by for profit status[44] | US | For profit: 0.11 | | | | | | Mean hours per resident per day |
|  |  | Non-profit: 0.11 | | | | | |  |
| Mean hours per resident per day of physiotherapy aide staffing by for profit status[44] | US | For profit: 0.02** | | | | | | Mean hours per resident per day |
|  |  | Non-profit: 0.03** (+) | | | | | |  |
| Mean hours per resident per day of occupational therapy staffing by for profit status[44] | US | For profit: 0.09** | | | | | | Mean hours per resident per day |
|  |  | Non-profit: 0.10** (+) | | | | | |  |
| Mean hours per resident per day of occupational therapy assistant staffing by for profit status[44] | US | For profit: 0.09** | | | | | | Mean hours per resident per day |
|  |  | Non-profit: 0.08** (+) | | | | | |  |
| Mean hours per resident per day of occupational therapy aide staffing by for profit status[44] | US | For profit: 0.01 | | | | | | Mean hours per resident per day |
|  |  | Non-profit: 0.01 | | | | | |  |
| ***Medicare/Medicaid certification*** | | | | | | | | |
| Percentage of residents that are Medicaid receiving physiotherapy services by staffing category (overall mean percentage 59.1%)[43] | US | High staffing:^c^ 46.8* (-) | | | | | | Percentage |
|  |  | Medium staffing:^c^ 63.7* (+) | | | | | |  |
|  |  | Low staffing:^c^ 66.3* (+) | | | | | |  |
| Percentage of residents that are Medicaid covered receiving physiotherapy assistant services by staffing category (overall mean percentage 59.1%)[43] | US | High staffing:^c^ 48.5* (-) | | | | | | Percentage |
|  |  | Medium staffing:^c^ 62.6* (+) | | | | | |  |
|  |  | Low staffing:^c^ 65.8* (+) | | | | | |  |
| Percentage of residents that are Medicaid covered receiving physiotherapy aide (overall mean percentage 59.1%)[43] | US | 56.8 | | | | | | Percentage |
| Percentage of residents that are Medicare covered receiving physiotherapy services by staffing category (overall mean percentage 15.2%)[43] | US | High staffing:^c^ 24.0* (+) | | | | | | Percentage |
|  |  | Medium staffing:^c^ 12.8* (-) | | | | | |  |
|  |  | Low staffing:^c^ 9.2* (-) | | | | | |  |
| Percentage of residents that are Medicare covered receiving physiotherapy assistant services by staffing category (overall mean percentage 15.2%)[43] | US | High staffing:^c^ 23.7* (+) | | | | | | Percentage |
|  |  | Medium staffing:^c^ 12.6* (-) | | | | | |  |
|  |  | Low staffing:^c^ 9.7* (-) | | | | | |  |
| Percentage of residents that are Medicare covered receiving physiotherapy aide (overall mean percentage 15.2%)[43] | US | 17.6 | | | | | | Percentage |
| Percentage of residents that are Medicaid covered receiving occupational therapy services by staffing category (overall mean percentage 59.1%)[43] | US | High staffing:^c^ 47.5* | | | | | | Percentage |
|  |  | Medium staffing:^c^ 63.3* (+) | | | | | |  |
|  |  | Low staffing:^c^ 66.1* (+) | | | | | |  |
| Percentage of residents that are Medicaid covered receiving occupational therapy assistant services by staffing category (overall mean percentage 59.1%)[43] | US | High staffing:^c^ 50.3* | | | | | | Percentage |
|  |  | Medium staffing:^c^ 63.4* (+) | | | | | |  |
|  |  | Low staffing:^c^ 63.1* (+) | | | | | |  |
| Percentage of residents that are Medicaid covered receiving occupational therapy aide (overall mean percentage 59.1%)[43] | US | 60.1 | | | | | | Percentage |
| Percentage of residents that are Medicare covered receiving occupational therapy services by staffing category (overall mean percentage 15.2%)[43] | US | High staffing:^c^ 24.0* (+) | | | | | | Percentage |
|  |  | Medium staffing:^c^ 12.9* (-) | | | | | |  |
|  |  | Low staffing:^c^ 9.2* (-) | | | | | |  |
| Percentage of residents that are Medicare covered receiving occupational therapy assistant services by staffing category (overall mean percentage 15.2%)[43] | US | High staffing:^c^ 22.8* (+) | | | | | | Percentage |
|  |  | Medium staffing:^c^ 12.7* (-) | | | | | |  |
|  |  | Low staffing:^c^ 10.6* (-) | | | | | |  |
| Percentage of residents that are Medicare receiving occupational therapy aide (overall mean percentage 15.2%)[43] | US | 16.4 | | | | | | Percentage |
| Likelihood of physiotherapy services in Medicaid certified facilities (reference group no Medicaid certification)[29] | US | 1.353** (1.094, 1.673) (+) | | | | | | Odds ratio (95% confidence interval) |
| Likelihood of occupational therapy services in Medicaid certified facilities (reference group no Medicaid certification)[29] | US | 1.276* (1.031, 1.578) (+) | | | | | | Odds ratio (95% confidence interval) |
| Percentage point change in use of qualified social service workers in Medicaid certified facilities (reference group not certified)[49] | US | -0.023** (0.010) (-) | | | | | | Coefficient estimates (standard error) |
| Percentage point change in use of paraprofessional to deliver social services in Medicaid certified facilities (reference group not certified)[49] | US | 0.016 (0.013) | | | | | | Coefficient estimates (standard error) |
| Percentage point change in use of interprofessional teams to deliver social services in Medicaid certified facilities (reference group not certified)[49] | US | -0.007 (0.011) | | | | | | Coefficient estimates (standard error) |
| Percentage point change in use of qualified social service workers in Medicare certified facilities (reference group not certified)[49] | US | 0.017 (0.012) | | | | | | Coefficient estimates (standard error) |
| Percentage point change in use of paraprofessional to deliver social services in Medicare certified facilities (reference group not certified)[49] | US | 0.019 (0.016) | | | | | | Coefficient estimates (standard error) |
| Percentage point change in use of interprofessional teams to deliver social services in Medicare certified facilities (reference group not certified)[49] | US | 0.036** (0.016) (+) | | | | | | Coefficient estimates (standard error) |
| Probability of receiving rehabilitation therapy in last month of life in Medicaid residents[53] | US | Low/medium therapy:^b^ 1.001 (0.002) | | | | | | Relative risk ratio (standard error) |
|  |  | High/ultrahigh therapy:^b^ 1.014** (0.004) (-) | | | | | |  |
| Probability of receiving rehabilitation therapy in last month of life in Medicare residents[53] | US | Low/medium therapy:^b^ 1.000 (0.003) | | | | | | Relative risk ratio (standard error) |
|  |  | High/ultrahigh therapy:^b^ 1.030** (0.006) (-) | | | | | |  |
| ***Service utilisation*** | | | | | | | | |
| Likelihood of physiotherapy services by personal care assistant hours per resident per day (reference group 0)[29] | US | <1 hours per resident per day: 2.668** (1.333, 5.342) (+) | | | | | | Odds ratio (95% confidence interval) |
|  |  | 1–1.999 hours per resident per day: 2.416* (1.229, 4.753) (+) | | | | | |  |
|  |  | 2–2.999 hours per resident per day: 2.668** (1.350, 5.274) (+) | | | | | |  |
|  |  | ≥3 hours per resident per day: 2.812** (1.454, 5.439) (+) | | | | | |  |
| Likelihood of occupational therapy services by personal care assistant hours per resident per day (reference group 0)[29] | US | <1 hours per resident per day: 3.713 ** (1.740, 7.925) (+) | | | | | | Odds ratio (95% confidence interval) |
|  |  | 1–1.999 hours per resident per day: 3.369** (1.602, 7.085) (+) | | | | | |  |
|  |  | 2–2.999 hours per resident per day: 4.209 ** (1.992, 8.894) (+) | | | | | |  |
|  |  | ≥3 hours per resident per day: 3.705 ** (1.791, 7.664) (+) | | | | | |  |
| Probability of receiving rehabilitation therapy in last month of life by registered nurse hours per resident per day[53] | US | Low/medium therapy:^b^ 0.855 (0.122) | | | | | | Relative risk ratio (standard error) |
|  |  | High/ultrahigh therapy:^b^ 0.525** (0.222) (+) | | | | | |  |
| Probability of receiving rehabilitation therapy in last month of life by licensed practical nurse hours per resident per day[53] | US | Low/medium therapy:^b^ 1.292* (0.089) | | | | | | Relative risk ratio (standard error) |
|  |  | High/ultrahigh therapy:^b^ 0.570** (0.169) (+) | | | | | |  |
| Probability of receiving rehabilitation therapy in last month of life by certified nurse assistant hours per resident per day[53] | US | Low/medium therapy:^b^ 0.896 (0.067) | | | | | | Relative risk ratio (standard error) |
|  |  | High/ultrahigh therapy:^b^ 1.175** (0.125) (-) | | | | | |  |
| Mean Resource Utilisation Group score by physiotherapy service staffing group (overall mean 1.2)[43] | US | High staffing:^c^ 1.2* (+) | | | | | | Mean |
|  |  | Medium staffing:^c^ 1.2* (+) | | | | | |  |
|  |  | Low staffing:^c^ 1.1* | | | | | |  |
| Mean Resource Utilisation Group score by physiotherapy assistant service staffing group (overall mean 1.2)[43] | US | High staffing:^c^ 1.2* (+) | | | | | | Mean |
|  |  | Medium staffing:^c^ 1.2* (+) | | | | | |  |
|  |  | Low staffing:^c^ 1.1* | | | | | |  |
| Mean Resource Utilisation Group score by physiotherapy aides (overall mean 1.2)[43] | US | 1.2* | | | | | | Mean |
| Mean Resource Utilisation Group score by occupational therapy service staffing group (overall mean 1.2)[43] | US | High staffing:^c^ 1.2* (+) | | | | | | Mean |
|  |  | Medium staffing:^c^ 1.2* (+) | | | | | |  |
|  |  | Low staffing:^c^ 1.1* | | | | | |  |
| Mean Resource Utilisation Group score by occupational therapy assistant service staffing group (overall mean 1.2)[43] | US | High staffing:^c^ 1.2* (+) | | | | | | Mean |
|  |  | Medium staffing:^c^ 1.2* (+) | | | | | |  |
|  |  | Low staffing:^c^ 1.1* | | | | | |  |
| Mean Resource Utilisation Group score by occupational therapy aides (overall mean 1.2)[43] | US | 1.2* | | | | | | Mean |
| ***Acuity*** | | | | | | | | |
| Mean acuity levels by physiotherapy service staffing group (overall mean 12.1)[43] | US | High staffing:^c^ 12.3* (+) | | | | | | Mean |
|  |  | Medium staffing:^c^ 12.3* (+) | | | | | |  |
|  |  | Low staffing:^c^ 11.9* | | | | | |  |
| Mean acuity levels by physiotherapy assistant service staffing group (overall mean 12.1)[43] | US | High staffing:^c^ 12.2* (+) | | | | | | Mean |
|  |  | Medium staffing:^c^ 12.2* (+) | | | | | |  |
|  |  | Low staffing:^c^ 11.9* | | | | | |  |
| Mean acuity levels by facilities which hired physiotherapy aides (overall mean 12.1)[43] | US | 12.3* (+) | | | | | | Mean |
| Mean acuity levels by occupational therapy service staffing group (overall mean 12.1)[43] | US | High staffing:^c^ 12.3* (+) | | | | | | Mean |
|  |  | Medium staffing:^c^ 12.3* (+) | | | | | |  |
|  |  | Low staffing:^c^ 11.9* | | | | | |  |
| Mean acuity levels by occupational therapy assistant service staffing group (overall mean 12.1)[43] | US | High staffing:^c^ 12.2* (+) | | | | | | Mean |
|  |  | Medium staffing:^c^ 12.2* (+) | | | | | |  |
|  |  | Low staffing:^c^ 11.9* | | | | | |  |
| Mean acuity levels by facilities which hired occupational therapy aides (overall mean 12.1)[43] | US | 12.2* (+) | | | | | | Mean |
| Percentage point change in use qualified social workers in facilities with high acuity index[49] | US | 0.001 (0.001) | | | | | | Coefficient estimates (standard error) |
| Percentage point change in use of paraprofessionals to deliver social services in facilities with high acuity index [49] | US | -0.001 (0.001) | | | | | | Coefficient estimates (standard error) |
| Percentage point change in use of interprofessional teams to deliver social services in facilities with high acuity index [49] | US | 0.000 (0.001) | | | | | | Coefficient estimates (standard error) |
| ***Hospital-based facilities*** | | | | | | | | |
| Percentage point change in use qualified social workers in hospital facilities (compared to free-standing facilities)[49] | US | 0.036* (0.018) (+) | | | | | | Coefficient estimates (standard error) |
| Percentage point change in use of paraprofessionals to deliver social services in hospital facilities (compared to free-standing facilities) [49] | US | -0.079** (0.022) (-) | | | | | | Coefficient estimates (standard error) |
| Percentage point change in use of interprofessional teams to deliver social services in hospital facilities (compared to free-standing facilities)[49] | US | -0.039 (0.022) (-) | | | | | | Coefficient estimates (standard error) |
| Mean hours per resident per day of physiotherapy services by hospital location[44] | US | Freestanding: 0.09** | | | | | | Mean hours per resident per day |
|  |  | Hospital-based: 0.20** (+) | | | | | |  |
| Mean hours per resident per day of physiotherapy assistant services by hospital location[44] | US | Freestanding: 0.11** | | | | | | Mean hours per resident per day |
|  |  | Hospital-based: 0.18** (+) | | | | | |  |
| Mean hours per resident per day of physiotherapy aide services by hospital location[44] | US | Freestanding: 0.02** | | | | | | Mean hours per resident per day |
|  |  | Hospital-based: 0.06** (+) | | | | | |  |
| Mean hours per resident per day of occupational therapy services by hospital location[44] | US | Freestanding: 0.09** | | | | | | Mean hours per resident per day |
|  |  | Hospital-based: 0.17** (+) | | | | | |  |
| Mean hours per resident per day of occupational therapy assistant services by hospital location[44] | US | Freestanding: 0.09** | | | | | | Mean hours per resident per day |
|  |  | Hospital-based: 0.12** (+) | | | | | |  |
| Mean hours per resident per day of occupational therapy aide services by hospital location[44] | US | Freestanding: 0.01** | | | | | | Mean hours per resident per day |
|  |  | Hospital-based: 0.02** (+) | | | | | |  |
| ***Occupancy rate*** | | | | | | | | |
| Likelihood of occupational therapy services by occupancy rate (reference group 1.0%–65.0%)[29] | US | 65.1%–80.0%: 0.889 (0.651, 1.214) | | | | | | Odds ratio (95% confidence interval) |
|  |  | 80.1%–95.0%: 0.868 (0.650, 1.160) | | | | | |  |
|  |  | 95.1%–100.0%: 1.031 (0.732, 1.453) | | | | | |  |
| Likelihood of physiotherapy services by occupancy rate (reference group 1.0%–65.0%)[29] | US | 65.1%–80.0%: 1.055 (0.774, 1.437) | | | | | | Odds ratio (95% confidence interval) |
|  |  | 80.1%–95.0%: 0.880(0.659, 1.177) | | | | | |  |
|  |  | 95.1%–100.0%: 1.064 (0.756, 1.498) | | | | | |  |
| Mean occupancy rate by physiotherapy service staffing group (overall mean 80.9)[43] | US | High staffing:^c^ 80.6* | | | | | | Mean |
|  |  | Medium staffing:^c^ 82.7* (+) | | | | | |  |
|  |  | Low staffing:^c^ 79.4* | | | | | |  |
| Mean occupancy rate by physiotherapy assistant service staffing group (overall mean 80.9)[43] | US | High staffing:^c^ 79.7* | | | | | | Mean |
|  |  | Medium staffing:^c^ 81.7* (+) | | | | | |  |
|  |  | Low staffing:^c^ 81.3* (+) | | | | | |  |
| Mean occupancy rate in facilities that hired facility physiotherapy aides (overall mean 80.9)[43] | US | 82.8* (+) | | | | | | Mean |
| Mean occupancy rate by occupational therapy service staffing group (overall mean 80.9)[43] | US | High staffing:^c^ 80.4* | | | | | | Mean |
|  |  | Medium staffing:^c^ 82.9* (+) | | | | | |  |
|  |  | Low staffing:^c^ 79.4* | | | | | |  |
| Mean occupancy rate by occupational therapy assistant service staffing group (overall mean 80.9)[43] | US | High staffing:^c^ 79.6* | | | | | | Mean |
|  |  | Medium staffing:^c^ 82.0* (+) | | | | | |  |
|  |  | Low staffing:^c^ 81.1* (+) | | | | | |  |
| Mean occupancy rate in facilities that hired facility occupational therapy aides (overall mean 80.9)[43] | US | 82.6* (+) | | | | | | Mean |
| Probability of therapy in last month of life by high occupancy rate[53] | US | Low/medium therapy:^b^ 1.001 (0.003) | | | | | | Relative risk ratio (standard error) |
|  |  | High/ultrahigh therapy:^b^ 0.995 (0.006) | | | | | |  |
| **Resident factors** | | | | | | | | |
| Probability of therapy in last month of life by age at death rate (reference group no therapy in last month of life)[53] | US | Low/medium therapy:^b^ 1.003 (0.002) | | | | | | Relative risk ratio (standard error) |
|  |  | High/ultrahigh therapy:^b^ 1.006 (0.003) | | | | | |  |
| Probability of therapy in last month of life in females (vs males) (reference group no therapy in last month of life)[53] | US | Low/medium therapy:^b^ 0.874** (0.039) | | | | | | Relative risk ratio (standard error) |
|  |  | High/ultrahigh therapy:^b^ 0.749** (0.053) | | | | | |  |
| Probability of therapy in last month of life in African Americans (vs white) (reference group no therapy in last month of life)[53] | US | Low/medium therapy:^b^ 0.814** (0.060) | | | | | | Relative risk ratio (standard error) |
|  |  | High/ultrahigh therapy:^b^ 1.069** (0.078) | | | | | |  |
| Probability of therapy in last month of life in married residents(reference group no therapy in last month of life)[53] | US | Low/medium therapy:^b^ 0.945 (0.043) | | | | | | Relative risk ratio (standard error) |
|  |  | High/ultrahigh therapy:^b^ 0.887* (0.060) | | | | | |  |
| Probability of therapy in last month of life in residents with at least one cardiovascular diagnosis (reference group no therapy in last month of life)[53] | US | Low/medium therapy:^b^ 1.097 (0.078) | | | | | | Relative risk ratio (standard error) |
|  |  | High/ultrahigh therapy:^b^ 1.431** (0.135) | | | | | |  |
| Probability of therapy in last month of life in residents with at least one gastrointestinal diagnosis (reference group no therapy in last month of life)[53] | US | Low/medium therapy:^b^ 0.996 (0.035) | | | | | | Relative risk ratio (standard error) |
|  |  | High/ultrahigh therapy:^b^ 1.109* (0.049) | | | | | |  |
| Probability of therapy in last month of life in residents with at least one genitourinary diagnosis (reference group no therapy in last month of life)[53] | US | Low/medium therapy:^b^ 1.088* (0.040) | | | | | | Relative risk ratio (standard error) |
|  |  | High/ultrahigh therapy:^b^ 1.084 (0.054) | | | | | |  |
| Probability of therapy in last month of life in residents with at least one infection diagnosis (reference group no therapy in last month of life)[53] | US | Low/medium therapy:^b^ 1.607** (0.034) | | | | | | Relative risk ratio (standard error) |
|  |  | High/ultrahigh therapy:^b^ 1.665** (0.047) | | | | | |  |
| Probability of therapy in last month of life in residents with diabetes (reference group no therapy in last month of life)[53] | US | Low/medium therapy:^b^ 0.980 (0.035) | | | | | | Relative risk ratio (standard error) |
|  |  | High/ultrahigh therapy:^b^ 0.983 (0.048) | | | | | |  |
| Probability of therapy in last month of life in residents with at least one other endocrine diagnosis (reference group no therapy in last month of life)[53] | US | Low/medium therapy:^b^ 1.215** (0.034) | | | | | | Relative risk ratio (standard error) |
|  |  | High/ultrahigh therapy:^b^ 1.113* (0.048) | | | | | |  |
| Probability of therapy in last month of life in residents with at least one musculoskeletal diagnosis (reference group no therapy in last month of life)[53] | US | Low/medium therapy:^b^ 1.224** (0.035) | | | | | | Relative risk ratio (standard error) |
|  |  | High/ultrahigh therapy:^b^ 1.343** (0.048) | | | | | |  |
| Probability of therapy in last month of life in residents with at least one neurologic diagnosis (reference group no therapy in last month of life)[53] | US | Low/medium therapy:^b^ 0.944 (0.051) | | | | | | Relative risk ratio (standard error) |
|  |  | High/ultrahigh therapy:^b^ 1.343** (0.048) | | | | | |  |
| Probability of therapy in last month of life in residents with malnutrition (reference group no therapy in last month of life)[53] | US | Low/medium therapy:^b^ 0.974 (0.079) | | | | | | Relative risk ratio (standard error) |
|  |  | High/ultrahigh therapy:^b^ 0.920 (0.123) | | | | | |  |
| Probability of therapy in last month of life in residents with at least psychiatric diagnosis (reference group no therapy in last month of life)[53] | US | Low/medium therapy:^b^ 1.076* (0.036) | | | | | | Relative risk ratio (standard error) |
|  |  | High/ultrahigh therapy:^b^ 1.035 (0.050) | | | | | |  |
| Probability of therapy in last month of life in residents with at least one pulmonary diagnosis (reference group no therapy in last month of life)[53] | US | Low/medium therapy:^b^ 1.101** (0.035) | | | | | | Relative risk ratio (standard error) |
|  |  | High/ultrahigh therapy:^b^ 1.057 (0.048) | | | | | |  |
| Probability of therapy in last month of life in residents with at least one vision diagnosis (reference group no therapy in last month of life)[53] | US | Low/medium therapy:^b^ 1.101** (0.038) | | | | | | Relative risk ratio (standard error) |
|  |  | High/ultrahigh therapy:^b^ 0.956 (0.054) | | | | | |  |
| Probability of therapy in last month of life by ADL score (0-28) (reference group no therapy in last month of life)[53] | US | Low/medium therapy:^b^ 1.001 (0.004) | | | | | | Relative risk ratio (standard error) |
|  |  | High/ultrahigh therapy:^b^ 0.983** (0.005) | | | | | |  |
| Probability of therapy in last month of life in residents who are mildly cognitively impaired (reference group no therapy in last month of life)[53] | US | Low/medium therapy:^b^ 1.128 (0.063) | | | | | | Relative risk ratio (standard error) |
|  |  | High/ultrahigh therapy:^b^ 1.228** (0.080) | | | | | |  |
| Probability of therapy in last month of life in residents who are moderately cognitively impaired (reference group no therapy in last month of life)[53] | US | Low/medium therapy:^b^ 1.213** (0.059) | | | | | | Relative risk ratio (standard error) |
|  |  | High/ultrahigh therapy:^b^ 1.257** (0.078) | | | | | |  |
| Probability of therapy in last month of life in residents who are severely cognitively impaired (reference group no therapy in last month of life)[53] | US | Low/medium therapy:^b^ 1.218** (0.065) | | | | | | Relative risk ratio (standard error) |
|  |  | High/ultrahigh therapy:^b^ 0.898 (0.088) | | | | | |  |
| Probability of therapy in last month of life in residents who were hospitalised in the two months before death (reference group no therapy in last month of life)[53] | US | Low/medium therapy:^b^ 3.206** (0.034) | | | | | | Relative risk ratio (standard error) |
|  |  | High/ultrahigh therapy:^b^ 8.182** (0.052) | | | | | |  |
| Likelihood of any physiotherapy in female residents[46] | Canada | 1.75** (1.24, 2.44) | | | | | | Adjusted odds ratio |
| Likelihood of any physiotherapy in residents with deteriorated health status (reference group stable)[46] | Canada | 0.97 (0.92, 1.03) | | | | | | Adjusted odds ratio |
| Likelihood of any physiotherapy in residents with improved health status (reference group stable)[46] | Canada | 1.97** (1.76, 2.21) | | | | | | Adjusted odds ratio |
| Likelihood of any physiotherapy in residents experiencing an acute episode (reference group stable)[46] | Canada | 1.14** (1.05, 1.25) | | | | | | Adjusted odds ratio |
| Likelihood of any physiotherapy in residents with self-rated potential to improve[46] | Canada | 1.30** (1.22, 1.39) | | | | | | Adjusted odds ratio |
| Likelihood of any physiotherapy in residents with potential to improve as rated by staff[46] | Canada | 1.16** (1.08, 1.25) | | | | | | Adjusted odds ratio |
| Likelihood of any physiotherapy in residents with any fracture[46] | Canada | 1.41** (1.32, 1.51) | | | | | | Adjusted odds ratio |
| Likelihood of any physiotherapy in residents with arthritis[46] | Canada | 1.09** (1.03, 1.14) | | | | | | Adjusted odds ratio |
| Likelihood of any physiotherapy in residents with osteoposis[46] | Canada | 1.14** (1.07, 1.21) | | | | | | Adjusted odds ratio |
| Likelihood of any physiotherapy in residents with stroke[46] | Canada | 1.33** (1.26, 1.41) | | | | | | Adjusted odds ratio |
| Likelihood of any physiotherapy in residents with multiple sclerosis[46] | Canada | 1.71** (1.48, 1.98) | | | | | | Adjusted odds ratio |
| Likelihood of any physiotherapy in residents with pneumoia[46] | Canada | 1.28** (1.17, 1.41) | | | | | | Adjusted odds ratio |
| Likelihood of any physiotherapy by activities of daily living hierarchy score[46] | Canada | 1.53** (1.45, 1.62) | | | | | | Adjusted odds ratio |
| Likelihood of any physiotherapy by cognitive performance scale[46] | Canada | 0.78** (0.74, 0.81) | | | | | | Adjusted odds ratio |
| Likelihood of any physiotherapy by depression rating scale[46] | Canada | 0.93** (0.91, 0.95) | | | | | | Adjusted odds ratio |
| Likelihood of any physiotherapy by pain scale score[46] | Canada | 1.18** (1.01, 1.27) | | | | | | Adjusted odds ratio |

*p<0.01; **p<0.05; ^a^ statistical significance not provided instead within the included study text the result was described as different or significant; ^b^ therapy levels in this study were defined as low (45-149 minutes), medium (150-324 minutes), high (325-499 minutes), very high (500-719 minutes), and ultrahigh (≥720 minutes); ^c^ In this study therapy levels were categorised as low (0–0.05 HPRD of physiotherapy, 0–0.06 HPRD of physiotherapy assistants, 0–0.05 HPRD of occupational therapy, 0–0.05 HPRD occupational therapy assistants), medium (0.05–0.10 HPRD of physiotherapy, 0.06– 0.12 HPRD of physiotherapy assistants, 0.05–0.10 HPRD of occupational therapy, 0.05–0.10 HPRD of occupational therapy assistants), and high (0.10–1.14 HPRD of physiotherapy, 0.12–9.04 HPRD of physiotherapy assistants, 0.10–0.91 HPRD of occupational therapy, 0.10–11.80 HPRD of occupational therapy assistants). Note: Positive association, as described in the text, is indicated by (+). Negative association is indicated by (-).
