# Supplementary material for: Therapy-based allied health delivery in residential aged care, trends, factors, and outcomes: a systematic review
Source: BMC Geriatr. 2022 Aug 28;22:712. doi: 10.1186/s12877-022-03386-9 (PMC9420184; doi:10.1186/s12877-022-03386-9)
Supplement: Supplementary file 5 — Additional file 5: Outcomes associated with allied health staffing. [file 12877_2022_3386_MOESM5_ESM.docx]

# Appendix 5

**Table 1.** Outcomes associated with allied health staffing

| **Outcome** | **Outcome measure tool** | **Effect** | **Measure** |
| --- | --- | --- | --- |
| ***Activities of daily living*** | | | |
| Association between individual frequency of physiotherapy at baseline and ADL score after six months[41] | 10 item minimum data set activities of daily living scale | -0.09** (0.04) (+) | Estimate (standard error) |
| Association between facility staffing levels (mean number of physiotherapists per ten beds) at baseline and ADL score after six months[41] | 10 item minimum data set activities of daily living scale | 0.08 (0.84) | Estimate (standard error) |
| Bivariate estimates for activities of daily living quality indicators and receiving no physiotherapy[47] | Activities of daily living hierarchy scale (ADL-H) | Late loss ADLs:^a^ 0.006 (-0.004, 0.016) | Estimate (95% confidence interval) |
|  |  | Mid-loss ADLs:^a^ -0.011 (-0.022, 0.000) |  |
|  |  | Early-loss ADLs:^a^ -0.0153 (-0.028, 0.003) |  |
| Bivariate estimates for activities of daily living quality indicator and receiving 45 minutes of physiotherapy >3 days[47] | Activities of daily living hierarchy scale (ADL-H) | Late loss ADLs:^a^ 0.008 (-0.001, 0.018) | Estimate (95% confidence interval) |
|  |  | Mid-loss ADLs:^a^ 0.008 (-0.003, 0.018) |  |
|  |  | Early-loss ADLs:^a^ -0.004 (-0.016, 0.008) |  |
| Bivariate estimates for activities of daily living quality indicators and receiving 45-150 minutes of physiotherapy 3-5 days[47] | Activities of daily living hierarchy scale (ADL-H) | Late loss ADLs:^a^ -0.006 (-0.014, 0.001) | Estimate (95% confidence interval) |
|  |  | Mid-loss ADLs:^a^ 0.003 (-0.005, 0.011) |  |
|  |  | Early-loss ADLs:^a^ 0.017** (0.007, 0.027) (+) |  |
| Bivariate estimates for activities of daily living quality indicators and receiving >150 minutes of physiotherapy >5 days[47] | Activities of daily living hierarchy scale (ADL-H) | Late loss ADLs:^a^ -0.158** (-0.227, 0.088) (+) | Estimate (95% confidence interval) |
|  |  | Mid-loss ADLs:^a^ 0.167 **(-0.243, - 0.091) (+) |  |
|  |  | Early-loss ADLs:^a^ 0.017** (-0.285, -0.117) (+) |  |
| Relationship between physiotherapy HPRD and ADLs measures[44] | four item MDS-ADL scale | 2.85** (0.94) (+) | Coefficient (robust standard error) |
| Relationship between physiotherapy assistant HPRD and ADLs measures[44] | four item MDS-ADL scale | 0.06 (0.58) | Coefficient (robust standard error) |
| Relationship between physiotherapy aide HPRD and ADLs measures[44] | four item MDS-ADL scale | 1.59 (1.22) | Coefficient (robust standard error) |
| Relationship between occupational therapy HPRD and ADLs measures[44] | four item MDS-ADL scale | 3.60** (0.97) (+) | Coefficient (robust standard error) |
| Relationship between occupational therapy assistant HPRD and ADLs measures[44] | four item MDS-ADL scale | -0.13 (0.47) | Coefficient (robust standard error) |
| Relationship between occupational therapy aide HPRD and ADLs measures[44] | four item MDS-ADL scale | -0.02 (0.45) | Coefficient (robust standard error) |
| ***Falls*** | | | |
| Bivariate estimates for falls in last 30 days and receiving no physiotherapy[47] | Falls in last 30 days | 0.010** (0.003, 0.016) (-) | Estimate (95% confidence interval) |
| Bivariate estimates for falls in last 30 days and receiving 45 minutes of physiotherapy >3 days[47] | Falls in last 30 days | 0.007 (0.000, 0.013) | Estimate (95% confidence interval) |
| Bivariate estimates for falls in last 30 days and receiving 45-150 minutes of physiotherapy 3-5 days[47] | Falls in last 30 days | -0.008** (-0.013, -0.004) (+) | Estimate (95% confidence interval) |
| Bivariate estimates for falls in last 30 days and receiving >150 minutes of physiotherapy >5 days[47] | Falls in last 30 days | -0.107** (-0.151, -0.063) (+) | Estimate (95% confidence interval) |
| Relationship between physiotherapy HPRD and falls measures[44] | Proportion of residents in facility who had a fall in last 30 days | 1.02** (0.28) (+) | Coefficient (robust standard error) |
| Relationship between physiotherapy assistant HPRD and falls measures[44] | Proportion of residents in facility who had a fall in last 30 days | -0.54** (0.18) (-) | Coefficient (robust standard error) |
| Relationship between physiotherapy aide HPRD and falls measures[44] | Proportion of residents in facility who had a fall in last 30 days | -0.57 (0.37) | Coefficient (robust standard error) |
| Relationship between occupational therapy HPRD and falls measures[44] | Proportion of residents in facility who had a fall in last 30 days | 0.94** (0.29) (+) | Coefficient (robust standard error) |
| Relationship between occupational therapy assistant HPRD and falls measures[44] | Proportion of residents in facility who had a fall in last 30 days | 0.20 (0.15) | Coefficient (robust standard error) |
| Relationship between occupational therapy aide HPRD and falls measures[44] | Proportion of residents in facility who had a fall in last 30 days | 0.02 (0.15) | Coefficient (robust standard error) |
| ***Care quality*** | | | |
| Likelihood of 5-Star quality measure by physiotherapy HPRD[44] | 5-star quality measure | 2.04* (0.63) (+) | Odds ratio (robust standard error) |
| Likelihood of 5-Star quality measure by physiotherapy assistant HPRD[44] | 5-star quality measure | 1.20 (0.24) | Odds ratio (robust standard error) |
| Likelihood of 5-Star quality measure by physiotherapy aide HPRD[44] | 5-star quality measure | 0.59 (0.24) | Odds ratio (robust standard error) |
| Likelihood of 5-Star quality measure by occupational therapy HPRD[44] | 5-star quality measure | 2.94** (0.97) (+) | Odds ratio (robust standard error) |
| Likelihood of 5-Star quality measure by occupational therapy assistant HPRD[44] | 5-star quality measure | 0.94 (0.16) | Odds ratio (robust standard error) |
| Likelihood of 5-Star quality measure by occupational therapy aide HPRD[44] | 5-star quality measure | 0.94 (0.21) | Odds ratio (robust standard error) |
| Likelihood of poorer seating outcomes by ratio of occupational therapy staff per resident[35] | SIT | 0.11** (0.04, 0.31) (+) | Odds ratio (95% confidence interval) |
| ***Nutrition*** | | | |
| Association between access to clinical/community dietician and being well nourished[50] | Mini Nutritional Assessment Short Form (MNA-SF) | 1.76* (1.07, 2.89) (+) | Odds ratio (95% confidence interval) |
| Association between access to Food service dietitian and being well nourished[50] | Mini Nutritional Assessment Short Form (MNA-SF) | 0.95 (0.56, 1.59) | Odds ratio (95% confidence interval) |
| Pearson’s product-moment correlations between access to clinical/community dietician and meal satisfaction[50] | Satisfaction survey | 0.440 ** (+) | Pearson’s product-moment correlations |
| Pearson’s product-moment correlations between access to food service dietician and meal satisfaction[50] | Satisfaction survey | 0.273 ** (+) | Pearson’s product-moment correlations |

*p<0.01, **p<0.05. ^a^ Early-loss ADLs include personal hygiene and dressing; mid-loss ADLs include: transfer, locomotion, and toilet use; late-loss ADLs include: bed mobility, transfer, eating, and toilet use. Note: Positive association, as described in the text, is indicated by (+). Negative association is indicated by (-).
